# A genetic interaction between RAP1 and telomerase reveals an unanticipated role for RAP1 in telomere maintenance

**DOI:** 10.1111/acel.12517

**Published:** 2016-09-01

**Authors:** Paula Martínez, Gonzalo Gómez‐López, David G. Pisano, Juana M. Flores, Maria A. Blasco

**Affiliations:** ^1^ Telomeres and Telomerase GroupMolecular Oncology ProgramSpanish National Cancer Centre (CNIO)Melchor Fernández Almagro 3MadridE‐28029Spain; ^2^ Bioinformatics Core UnitStructural Biology and Biocomputing ProgramSpanish National Cancer Centre (CNIO)Melchor Fernández Almagro 3MadridE‐28029Spain; ^3^ Animal Surgery and Medicine DepartmentFaculty of VeterinarianComplutense University of MadridMadrid28029Spain

**Keywords:** premature aging, RAP1, shelterins, telomeres, telomerase

## Abstract

RAP1 is one of the components of shelterin, the capping complex at chromosome ends or telomeres, although its role in telomere length maintenance and protection has remained elusive. RAP1 also binds subtelomeric repeats and along chromosome arms, where it regulates gene expression and has been shown to function in metabolism control. Telomerase is the enzyme that elongates telomeres, and its deficiency causes a premature aging in humans and mice. We describe an unanticipated genetic interaction between RAP1 and telomerase. While RAP1 deficiency alone does not impact on mouse survival, mice lacking both RAP1 and telomerase show a progressively decreased survival with increasing mouse generations compared to telomerase single mutants. Telomere shortening is more pronounced in *Rap1* ^−/−^ *Terc* ^−/−^ doubly deficient mice than in the single‐mutant *Terc* ^−/−^ counterparts, leading to an earlier onset of telomere‐induced DNA damage and degenerative pathologies. Telomerase deficiency abolishes obesity and liver steatohepatitis provoked by RAP1 deficiency. Using genomewide ChIP sequencing, we find that progressive telomere shortening owing to telomerase deficiency leads to re‐localization of RAP1 from telomeres and subtelomeric regions to extratelomeric sites in a genomewide manner. These findings suggest that although in the presence of sufficient telomere reserve RAP1 is not a key factor for telomere maintenance and protection, it plays a crucial role in the context of telomerase deficiency, thus in agreement with its evolutionary conservation as a telomere component from yeast to humans.

## Introduction

Mammalian telomeres are composed of tandem repeats of the TTAGGG sequence bound by a specialized protein complex known as shelterin, which protects chromosome ends and regulates telomerase activity (Takai *et al*., 2003; de Lange, 2005; Martinez & Blasco, 2010, 2011). Telomere repeats can extend to different lengths in different species, and this length can also vary depending on the developmental stage and cell type within a given species (Flores *et al*., 2008; Marion *et al*., 2009). During each cell division cycle, telomeres shorten as a result of the incomplete replication of linear DNA molecules by conventional DNA polymerases, the so‐called ‘end‐replication problem’ (Watson, 1972; Olovnikov, 1973). Telomerase is a reverse transcriptase (TERT) capable of compensating telomere attrition through *de novo* addition of TTAGGG repeats onto the chromosome ends using an associated RNA component as template (*Terc*) (Greider & Blackburn, 1985). Telomerase is expressed in embryonic stem cells and in most adult stem cell compartments; however, this is not sufficient to maintain telomere length, and therefore, telomere shortening takes place with age in most tissues (Harley *et al*., 1990; Liu *et al*., 2007; Flores *et al*., 2008). This progressive telomere shortening has been proposed as one of the molecular hallmarks of aging (Lopez‐Otin *et al*., 2013). Indeed, longevity is progressively decreased upon successive intercrossing of *Terc*‐deficient mice, which is concomitant with a decreased mobilization of adult stem cell populations and premature organ failure. In this regard, *Terc*‐deficient mice show aging‐associated pathologies such as alopecia, lower body weight, intestinal atrophy, hair graying, infertility, heart dysfunction, bone marrow aplasia, kidney dysfunction, defective bone marrow, and proliferative defects of neural stem cells (Lee *et al*., 1998; Herrera *et al*., 1999; Samper *et al*., 2002; Blasco, 2005; Garcia‐Cao *et al*., 2006). Telomerase‐deficient mice are resistant to cancer, with the only exception of p53‐deficient and TRF2‐overexpressing genetic backgrounds (Artandi *et al*., 2000; Blanco *et al*., 2007).

RAP1 is one of the shelterin complexes that protects the telomeres (for a review, see de Lange, 2005; Martinez & Blasco, 2010, 2011). RAP1 binds to telomeric repeats through its interaction with TRF2 (Li *et al*., 2000). Human cell lines lacking RAP1 do not show either a DNA damage response (DDR) or telomere fusions except in the absence of TRF2 when RAP1 has a role in inhibition of the NHEJ pathway at telomeres (Sarthy *et al*., 2009). The role of human RAP1 in telomere length regulation is unclear, while some works have reported no telomere length changes in the absence of RAP1, others have observed telomere lengthening upon both RAP1 knockdown and RAP1 overexpression (Li *et al*., 2000; O'Connor *et al*., 2004; Sarthy *et al*., 2009; Kabir *et al*., 2014). Mouse cells lacking RAP1 have a moderate increase in fragile telomeres and in telomeric sister chromatid exchange events but are protected against telomere fusions (Martinez *et al*., 2010; Sfeir *et al*., 2010). Thus, in contrast to yeast where a crucial role of RAP1 in telomere length regulation and telomere protection has unequivocally been demonstrated (Lustig *et al*., 1990; Sussel & Shore, 1991; Kyrion *et al*., 1992; Kanoh & Ishikawa, 2001; Pardo & Marcand, 2005), human and mouse RAP1 does not seem to have an important function in telomere protection. Mammalian RAP1 and their unicellular orthologs have been shown to affect gene expression, and this transcriptional regulatory function has been proposed to reflect the molecular conservation among RAP1 proteins (Kabir *et al*., 2014).

RAP1 also associates to nontelomeric genomic sites where it has been demonstrated to regulate gene expression (Sarthy *et al*., 2009; Martinez *et al*., 2010, 2013; Yang *et al*., 2011; Yeung *et al*., 2013). Mice lacking RAP1 are viable but develop obesity, glucose intolerance, and hepatic steatosis. These signs of metabolic disorders are more pronounce in females than in males (Martinez *et al*., 2013; Yeung *et al*., 2013). Gene expression profile analyses in liver of adult mice revealed that in the absence of RAP1, several metabolic pathways are remarkably affected (Martinez *et al*., 2013).

If mammalian RAP1 is dispensable for telomere protection, why has RAP1 been conserved as a mayor telomere‐binding protein in mammals? Given its transcriptional regulatory role, it is possible that RAP1 has been conserved as a shelterin component to sense changes in telomeric states and to coordinate the regulation of different signaling pathways involved in different biological functions such as metabolism and DNA repair. To address the potential RAP1 role in the response to short telomeres and to determine whether RAP1 deficiency could rescue the low body weight presented by late‐generation telomerase knockout mice, we generated the double knockout *Rap1* ^−/−^ *Terc* ^−/−^ mouse model. We found that mice lacking both RAP1 and telomerase show a progressive decreased survival with increasing mouse generations as compared to telomerase single mutants, demonstrating an unanticipated genetic interaction between RAP1 and telomerase. Telomere shortening was more pronounced in *Rap1* ^−/−^ *Terc* ^−/−^ than in *Terc* ^−/−^ counterparts, leading to an earlier onset of DNA damage and its consequent DDR causing an anticipation of degenerative pathologies in the intestines. In its turn, telomerase deficiency abolishes RAP1‐mediated obesity and liver pathologies associated with metabolic syndrome. Cells with shortened telomeres present less amount of telomere‐bound RAP1 but show higher numbers of RAP1‐bound extratelomeric sites genomewide. Our findings demonstrate that although RAP1 is not a key factor in telomere capping under normal conditions, under certain cellular stresses such as telomerase deficiency, RAP1 exerts an important function for telomere length regulation and protection. These unanticipated findings provide a likely explanation for the fact that RAP1 is the most evolutionarily conserved shelterin.

## Results

### RAP1 abrogation exacerbates loss of organismal survival associated with short telomeres

**Figure 1:**
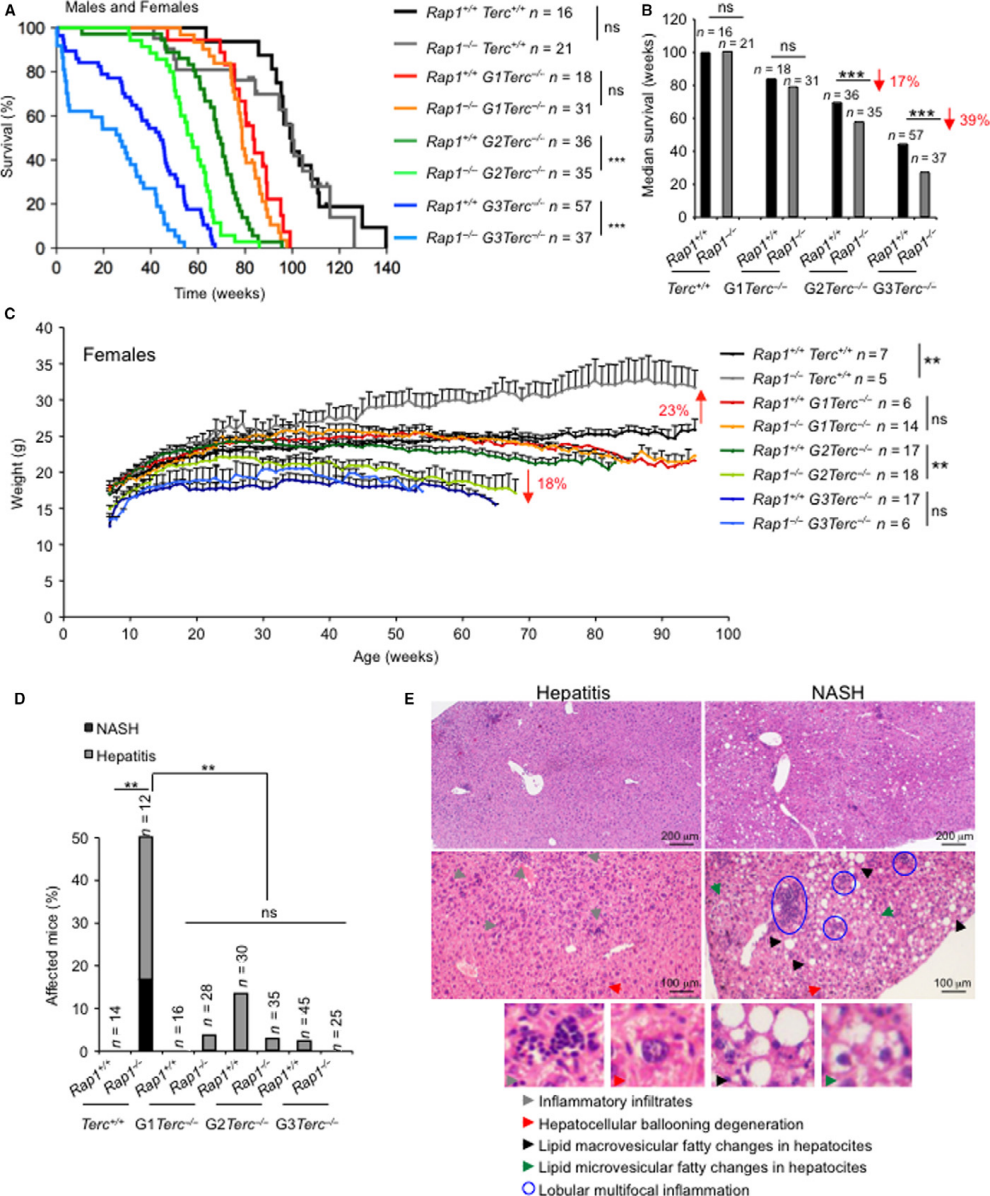
RAP1 loss decreases survival of telomerase‐deficient mice, and telomerase deficiency abolishes RAP1‐mediated obesity. (A) Kaplan–Meyer survival curves and (B) median survival from Kaplan–Meyer plots of *Rap1* ^+/+^ *Terc* ^+/+^, *Rap1* ^−/−^ *Terc* ^+/+^, G1 *Rap1* ^+/+^ *Terc* ^−/−^, G1 *Rap1* ^−/−^ *Terc* ^−/−^, G2 *Rap1* ^+/+^ *Terc* ^−/−^, G2 *Rap1* ^−/−^ *Terc* ^−/−^, G3 *Rap1* ^+/+^ *Terc* ^−/−^, and G3 *Rap1* ^−/−^ *Terc* ^−/−^ mice from both genders. The percent decrease in median survival in successive generations (G2 and G3) of *Rap1* ^−/−^ *Terc* ^−/−^ compared to *Rap1* ^+/+^ *Terc* ^−/−^ mice is shown (red arrows). Statistical comparisons among genotypes using the log‐rank test (A) and Student's *t*‐test (B) are shown. (C) Body weight curves of *Rap1* ^+/+^ *Terc* ^+/+^, *Rap1* ^−/−^ *Terc* ^+/+^, G1 *Rap1* ^+/+^ *Terc* ^−/−^, G1 *Rap1* ^−/−^ *Terc* ^−/−^, G2 *Rap1* ^+/+^ *Terc* ^−/−^, G2 *Rap1* ^−/−^ *Terc* ^−/−^, G3 *Rap1* ^+/+^ *Terc* ^−/−^, and G3 *Rap1* ^−/−^ *Terc* ^−/−^ female mice. The percent weight changes in *Rap1* ^−/−^ *Terc* ^+/+^ and in G2 *Rap1* ^−/−^ *Terc* ^−/−^ compared to their *Rap1* ^+/+^ counterparts at end point are indicated (red arrows). Values and error bars represent the mean and standard error, respectively. Statistical significances were calculated by the Student's t‐test. (D) Incidence of liver disease at time of death of mice of the indicated genotype. Statistical significances were calculated by the chi‐squared test. (E) Representative light microscopy images of hematoxylin‐/eosin‐stained sections of liver diagnosed for hepatitis (left panels) and nonalcoholic steatohepatitis (NASH) (right panels). Upper and lower panels correspond to low‐ and high‐magnification images, respectively. Scale bars are shown. Examples of inflammatory infiltrates, hepatocellular ballooning degeneration, and macro‐ and microvesicular depots are shown in high‐magnification inserts. Hepatitis is characterized as lobular inflammation with lymphocytes, macrophages and neutrophiles infiltrates and hepatocellular ballooning degeneration. NASH is a progressive process that begins with lipid deposition in the liver, showing both macro‐ and microvesicular fatty changes in hepatocyte's cytoplasm followed by hepatocellular ballooning degeneration and lobular multifocal inflammation. (*n*) Number of mice of each genotype used in the analysis. **, *p*<0.01, ***p<0.001, ns: no significant.

To address a putative role of RAP1 in the organismal response to short telomeres, we generated a *Rap1* ^−/−^ *Terc* ^−/−^ double‐mutant mouse by crossing the *Rap1* ^−/−^ with the *Terc* ^−/−^ mice (Blasco *et al*., 1997; Martinez *et al*., 2013). The resulting double heterozygous breeding pairs were used to generate first generation (G1) of single *Terc* ^−/−^ and of double *Terc* ^−/−^ *Rap1* ^−/−^. Large colonies of increasing *Terc* ^−/−^ mouse generations up to the third generation (G3) were generated by intercrossing G1 and G2 *Rap1* ^+/−^ *Terc* ^−/−^ pairs. Survival curves were monitored for the different mouse cohorts under study. As shown in Fig. 1A,B, increasing generations of G1‐G3 *Terc* ^−/−^ mice showed a progressive decrease in both median survival and mean lifespan compared to telomerase WT controls, in agreement with previous findings showing a premature loss of mouse survival associated with short telomeres (Herrera *et al*., 1999; Garcia‐Cao *et al*., 2006). *Rap1* deletion did not impact in mouse survival as shown by similar survival curves for single *Rap1* ^−/−^ mutant mice and *Rap1* ^+/+^ WT controls (Martinez *et al*., 2013; Yeung *et al*., 2013). Unexpectedly, simultaneous lack of telomerase and RAP1 in G1‐G3 *Terc* ^−/−^/*Rap1* ^−/−^ mice resulted in decreased survival compared to the RAP1‐proficient controls, a difference that reached statistical significance for the late‐generations G2 and G3 (Fig. 1A,B). This effect was gender‐independent as it was observed both in males and females (Fig. S1A–D, Supporting information). These results suggest that RAP1 deficiency aggravates the telomerase‐associated loss of organismal survival.

### Telomerase deficiency abolishes RAP1‐mediated obesity and liver pathologies

Whole‐body *Rap1* deletion results in obese mice, being this phenotype more severe in RAP1‐deficient females, while telomerase‐deficient mice have been shown to present lower body weight than WT mice, most likely owing to intestinal degenerative pathologies (Lee *et al*., 1998; Herrera *et al*., 1999; Martinez *et al*., 2013; Yeung *et al*., 2013). To assess the effects of double RAP1 and telomerase deficiencies on fat accumulation, we weekly followed body weight from mice of both genders of the different genotypes under study, *Rap1* ^+/+^ *Terc* ^+/+^, *Rap1* ^−/−^ *Terc* ^+/+^, G1‐G3 *Rap1* ^+/+^ *Terc* ^−/−^, and G1‐G3 *Rap1* ^−/−^ *Terc* ^−/−^ (Figs 1C and S1E, Supporting information). RAP1‐deficient females showed a progressive increase in body weight with time from week 8 onwards, reaching an approximately 25% increase in body weight compared to WT females at 1 year of age (Fig. 1C). The increased body weight in *Rap1*‐deficient females was maintained throughout their lifespan (Fig. S1F, Supporting information). Remarkably, female obesity owing to *Rap1* deficiency was abrogated in G1‐G3 *Rap1* ^−/−^ *Terc* ^−/−^ compound mice and even reversed in G2 *Rap1* ^−/−^ *Terc* ^−/−^ where a significant lower body weight was observed as compared to G2 *Rap1* ^+/+^ *Terc* ^−/−^ females (Fig. 1C). We did not observed significant differences in body weight among the different RAP1‐proficient and RAP1‐deficient male cohorts (Fig. S1E, Supporting information), in contrast to the moderate increase in body weight previously observed in a different *Rap1*‐deficient mouse cohort (Martinez *et al*., 2013). This difference in the impact of *Rap1* deficiency in male body weight might be explained by slight differences in genetic background associated with generation of the *Rap1 Terc* compound mouse colony.

We previously reported that Rap1‐deficient mice developed liver pathologies reminiscent to those associated with metabolic syndrome, that is, hepatic steatosis and inflammation (Martinez *et al*., 2013). To address the impact of telomerase deficiency and short telomeres in these pathologies, we performed histopathological analysis of the liver in all the mouse cohorts under study at their time of death. As expected, 50% of *Rap1* ^−/−^ *Terc* ^+/+^ male and female mice presented liver pathologies that were classified as either severe multifocal hepatitis or as nonalcoholic steatohepatitis (NASH) (Fig. 1D,E) (see Appendix S1, Supporting information). Importantly, no liver pathologies with the exception of some few cases of inflammation associated with aged liver were observed in telomerase‐deficient mice regardless of *Rap1* genotype (Fig. 1D). These results indicate that RAP1‐mediated liver pathologies are abrogated in the context of telomerase‐deficient mice.

### RAP1 abrogation leads to an earlier onset of aging pathologies associated with short telomeres

**Figure 2:**
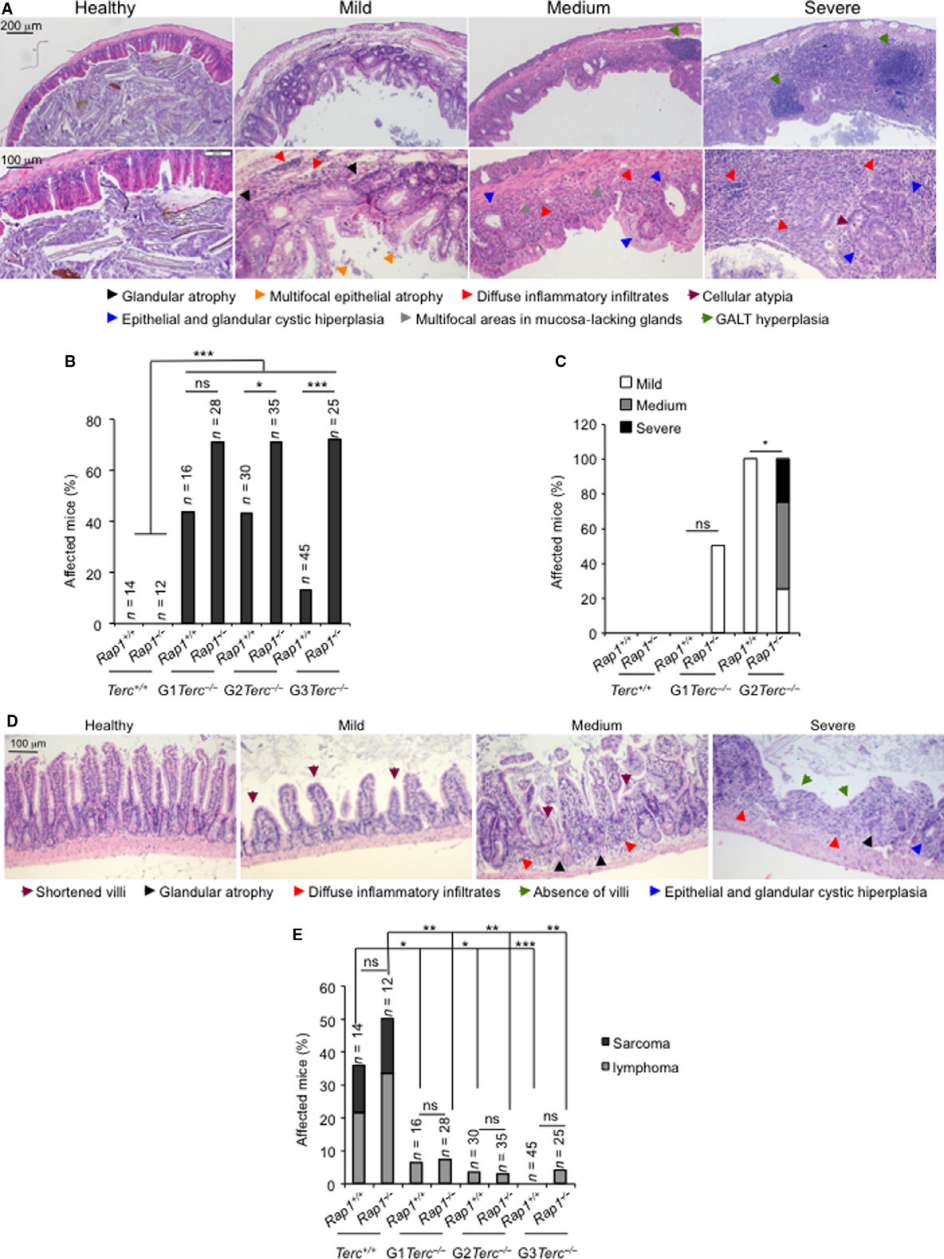
RAP1 loss aggravates intestinal degenerative pathologies of telomerase‐deficient mice. (A) Representative hematoxylin and eosin images of degenerative lesions of the intestine. Upper and lower panels correspond to low‐ and high‐magnification images. Scale bars are shown. Lesions were classified as mild, medium, and severe lesions according to the pathological findings. (B) Percentage of mice of the indicated genotypes showing severe intestinal atrophy at the time of death. (C) Percentage of mice of the indicated genotypes showing mild, medium, or severe intestinal atrophy at time point within their health span period during which animals remain healthy with no pathological signs. *Rap1*‐WT and *Rap1*‐null telomerase proficient were sacrificed at 25–50 week of age and G1‐G2 telomerase‐deficient mice at 22–30 week of age. (D) Representative hematoxylin and eosin images of degenerative and inflammatory lesions of the intestine of healthy young mice. (E) Percentage of mice of the indicated genotype showing malignant tumors (lymphoma and sarcomas) at their time of death. Statistical comparisons in B, C, and E between genotypes using the chi‐squares test are shown. (*n*) Number of mice of each genotype used in the analysis. *p<0.05; **, *p*<0.01, ***p<0.001, ns: no significant.

To determine the cause of death in the different mouse cohorts, we performed a full histopathological analysis in all the mice at their end point. Atrophies of the intestinal epithelia are the most frequent and life‐threatening degenerative lesions associated with short telomeres in G1‐G3 *Terc* ^−/−^ mice, which contribute to the early mortality of these mice (Herrera *et al*., 1999; Garcia‐Cao *et al*., 2006; Martinez *et al*., 2009a). We classified these intestinal pathologies in mild, medium, or severe according to the pathological findings (see Appendix S1, Supporting information) (Fig. 2A). Severe intestinal atrophy was absent in WT and single *Rap1*‐deficient mice at their time of death (Fig. 2B). In contrast, telomerase‐deficient mice showed severe intestinal mucosal lesions characterized by epithelial and glandular atrophy and inflammatory reactions (Fig 2B). Simultaneous telomerase and RAP1 deficiencies further increased the frequency of severe lesions, reaching statistical significance in G2‐G3 *Rap1* ^−/−^ *Terc* ^−/−^ compared to the G2‐G3 *Rap1* ^+/+^ *Terc* ^−/−^ counterparts (Fig. 2A,B). These observations are in line with the decreased survival observed in G2‐G3 *Rap1* ^−/−^ *Terc* ^−/−^ compared with their *Rap1* ^+/+^ counterparts. Histopathological analysis of kidneys, lungs, spleen, and heart did not reveal any significant differences between G0‐G3 *Rap1* ^+/+^ and G0‐G3 *Rap1* ^−/−^ mice (Fig. S2A, Supporting information).

As the above results were obtained at the time of death, to confirm that RAP1 deficiency aggravates degenerative pathologies at the GI track of *Terc* ^−/−^ mice, we sacrificed a group of 4–5 healthy young mice per group before any pathological signs at the age of 40–50 and 22–28 week old in G0 and G1‐G3 groups, respectively. Histopathological analysis of the GI track revealed that intestinal pathologies observed in mice showed already generalized degenerative intestinal lesions as well as inflammation, while none of the G2‐*Rap1* ^+/+^ *Terc* ^−/−^ mice showed these lesions in a similar age range. Instead, the G2‐*Rap1* ^+/+^ *Terc* ^−/−^ animals only showed mild focal lesions. No lesions were observed either in *Rap1* ^+/+^ *Terc* ^+/+^, *Rap1* ^−/−^ *Terc* ^+/+^ or in G1‐*Rap1* ^+/+^ *Terc* ^−/−^, while 50% of the G1‐*Rap1* ^−/−^ *Terc* ^−/−^ mice already showed mild atrophy (Fig. 2C,D) (see Appendix S1, Supporting information). Thus, RAP1 deficiency accelerates the onset of severe intestinal atrophy in late‐generation telomerase‐deficient mice. Histopathological analysis of the liver in these mice revealed that 75% of the *Rap1* ^−/−^ mice already present signs of hepatosteatosis, while none of the G2‐*Rap1* ^−/−^ *Terc* ^−/−^ showed this pathology (Fig. S2B, Supporting information).

**Figure 3:**
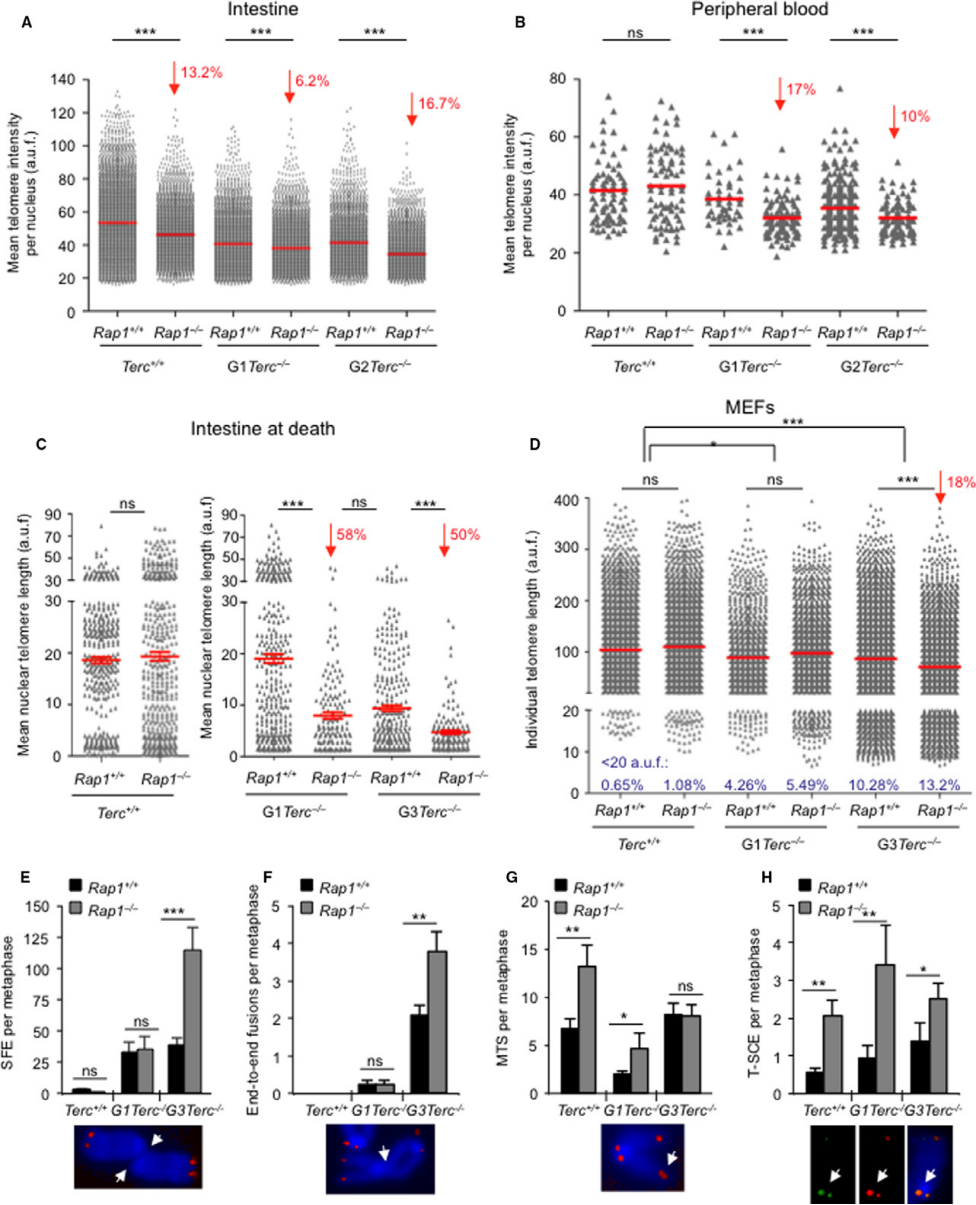
RAP1 loss leads to a decrease in telomere length in proliferative tissues of telomerase‐deficient mice. (A, B) Mean telomere fluorescence intensity distribution in small intestine sections (A) and in peripheral blood (B) of healthy young mice of the indicated genotype as determined by Q‐FISH analysis. (C). Mean nuclear telomere fluorescence intensity in small intestine sections of mice of the indicated genotype at death. (*n*) Four to five mice per genotype were analyzed. (D) Individual telomere length fluorescence intensity in metaphase spreads of immortalized MEFs of the indicated genotypes. (*n*) Two independent MEFs per genotype. Mean values (red lines) and percent telomere length decrease in *Rap1* ^−/−^ as compared to *Rap1* ^+/+^ in each group (red arrows) are indicated. The percent of critically short telomeres below percentile 5 is shown. (E, F, G, H) Incidence of signal‐free ends (SFE) (E), end‐to‐end fusions (F), multitelomeric signals (MTS) (G), and telomeric sister chromatid exchange (T‐SCE) (H) events in metaphase spreads of immortalized MEFs of the indicated genotypes. A representative image of each chromosomal aberration is shown below. (*n*) Two independent MEFs per genotype. The Student's *t*‐test was used for statistical analysis in each case. *p<0.05; **, *p*<0.01, ***p<0.001, ns: no significant.

We next analyzed the presence of malignant tumors (i.e. lymphomas and sarcomas) at the time of death of the different mouse cohorts. RAP1 deficiency did not lead to increased incidence of spontaneous malignant tumors (Fig. 2E). Telomerase‐deficient mice presented a drastically reduced incidence in malignant tumors at their time of death compared to *Terc* ^+/+^ mice (Fig. 3E), in agreement with a potent tumor suppressor activity of short telomeres (Gonzalez‐Suarez *et al*., 2000). This tumor protection conferred by telomerase deficiency was not altered in the absence of RAP1 (Fig. 2E).

### RAP1 loss leads to a more pronounce telomere shortening in proliferative tissues in TERC‐deficient mice

The anticipation and increased severity of degenerative GI pathologies in *Terc Rap1* doubly deficient mice suggest a role of RAP1 in telomere maintenance, which is unveiled in the absence of telomerase. In this regard, previous analysis of single RAP1‐deficient mice suggested that RAP1 was largely dispensable for telomere maintenance *in vivo* (Martinez *et al*., 2010, 2013).

We set to address the impact of *Rap1* deletion in telomere length maintenance *in vivo* in various mouse tissues in the context telomerase deficiency. To this end, we performed telomere quantitative FISH (Q‐FISH) analysis on tissue sections of intestine, liver, and of gelatine‐embedded peripheral white blood cells (PWBC) from mice of the different genotypes before any pathological signs (same mice as in Fig. 2C,D). Telomere length was moderately but significantly decreased in the RAP1‐deficient intestines compared with their RAP1‐proficient counterparts, regardless of telomerase status (Fig. 3A). Similarly, we detected a shorter mean telomere length in peripheral white blood cells (PWBC) from G1‐G2 *Rap1* ^−/−^ *Terc* ^−/−^ compared with their G1‐G2 *Rap1* ^+/+^ *Terc* ^−/−^ counterparts, while no differences in telomere length were detected between *Rap1* ^−/−^ *Terc* ^+/+^ and *Rap1* ^+/+^ *Terc* ^+/+^ cohorts (Fig. 3B). In the case of a nonproliferative tissue, such as the liver, however, RAP1 deficiency did not affect mean telomere length either in telomerase WT or in G2 *Terc* ^−/−^ livers (Fig. S2C, Supporting information). These results indicate a role for RAP1 in telomere maintenance in highly proliferative tissues in the context of telomerase deficiency.

We next performed Q‐FISH analysis on intestine sections at the time of death in the different mouse cohorts. No significant differences in nuclear telomere length were detected when comparing *Rap1* ^+/+^ *Terc* ^+/+^ and to *Rap1* ^−/−^ *Terc* ^+/+^ samples (Fig. 3C). Strikingly, the mean nuclear telomere length of G1‐*Rap1* ^−/−^ *Terc* ^−/−^ was dramatically decreased compared with G1‐*Rap1* ^+/+^ *Terc* ^−/−^, reaching values similar to those observed in G3‐*Rap1* ^+/+^ *Terc* ^−/−^ mice (Fig. 3C). Similarly, the telomere length of G3‐*Rap1* ^−/−^ *Terc* ^−/−^ intestines was significantly reduced compared to G3‐*Rap1* ^+/+^ *Terc* ^−/−^. Thus, mean telomere length in both G1 and G3 *Rap1* ^−/−^ *Terc* ^−/−^ was reduced by 50% compared with G1‐G3 *Rap1* ^+/+^ *Terc* ^−/−^ controls. Note that acquisition settings used for microscopy image capture were different in *Terc* ^+/+^ and *Terc* ^−/−^ samples because the settings used in *Terc* ^−/−^ samples resulted in saturated detention levels in *Rap1* ^+/+^ *Terc* ^+/+^ and to *Rap1* ^−/−^ *Terc* ^+/+^ samples (Fig. 3C). Altogether, these data indicate that RAP1 suppresses further telomere shortening associated with tissue homeostasis and aging in the setting of telomerase‐deficient mice, thus unveiling an important role for RAP1 in *in vivo* telomere length regulation.

Finally, we analyzed telomere length in mouse embryonic fibroblasts isolated from the different generation cohorts studied here. To this end, we first established two independent primary embryonic fibroblasts (MEFs) from each genotype under study that we later immortalized by retroviral transduction with SV40 large T (LT) antigen. We carried out Q‐FISH on metaphase spreads of immortalized *Rap1* ^+/+^ *Terc* ^+/+^, *Rap1* ^−/−^ *Terc* ^+/+^, G1 *Rap1* ^+/+^ *Terc* ^−/−^, G1 *Rap1* ^−/−^ *Terc* ^−/−^, G3 *Rap1* ^+/+^ *Terc* ^−/−^, and G3 *Rap1* ^−/−^ *Terc* ^−/−^ MEFs at passage 10 (Fig. 3D). Deletion of *Rap1* did not result in significant differences in mean telomere length between either *Rap1* ^+/+^ *Terc* ^+/+^ and *Rap1* ^−/−^ *Terc* ^+/+^ or between G1 *Rap1* ^+/+^ *Terc* ^−/−^ and G1 *Rap1* ^−/−^ *Terc* ^−/−^, although a modest increase in the percent of critically short telomeres (below percentile 5) was revealed (Fig. 3D). However, G3 *Rap1* ^−/−^ *Terc* ^−/−^ MEFs showed a 18% decrease in telomere fluorescence when compared to their WT controls, which was accompanied by an 3% increase in the frequency of critically short telomeres (below percentile 5). In addition, the number of signal‐free ends (SFE) per metaphase was significantly increased by 3‐fold in G3 *Rap1* ^−/−^ *Terc* ^−/−^ as compared to their *Rap1* ^+/+^ counterparts (Fig. 3E). These results further confirm a role for RAP1 in telomere length maintenance in the setting of telomerase deficiency.

### RAP1 abrogation induces a more severe telomere uncapping in late‐generation Terc‐deficient MEFs

Our findings indicate that mice and MEFs lacking RAP1 experience a faster and enhanced telomere shortening in the absence of telomerase. However, a putative role for RAP1 in facilitating telomerase action on chromosome ends is ruled out by our observation that RAP1‐mediated telomere erosion is particularly evident in a telomerase‐deficient background. Thus, the role of RAP1 in telomere length homeostasis must rather be due to a protective function that is dispensable when telomeres are long but becomes relevant when telomeres are short.

To address this, we performed cytogenetics analysis on metaphase spreads of the immortalized MEFs described above (Fig. 3F–G). In agreement with higher numbers of SFE, we also observed a 2‐fold increase in the number of end‐to‐end fusion events per metaphase in G3 *Rap1* ^−/−^ *Terc* ^−/−^ compared with G3 *Rap1* ^+/+^ *Terc* ^−/−^ MEFs, while no differences were detected between *Rap1* ^−/−^ *Terc* ^+/+^ and *Rap1* ^+/+^ *Terc* ^+/+^ or between G1 *Rap1* ^−/−^ *Terc* ^−/−^ and G1 *Rap1* ^+/+^ *Terc* ^−/−^ MEFs (Fig. 3F). As previously reported by us (Martinez *et al*., 2010), *Rap1* deletion induces a moderate increase in the number of multitelomeric signals (MTS), a telomere aberration that is indicative of telomere fragility (Martinez *et al*., 2009b), both in *Terc* ^+/+^ and in G1‐*Terc* ^−/−^ MEFs. This effect was abolished in G3‐*Terc* ^−/−^ probably due to the presence of high numbers of SFE (Fig. 3G). In accordance with a RAP1 role in prevention of homologous recombination at telomeres (Martinez *et al*., 2010; Sfeir *et al*., 2010), we also detected a significant increase in sister telomere recombination (T‐SCE) frequencies in (G0‐G1‐G3) *Rap1‐*deleted MEFs compared to *Rap1* WT controls as determined by the CO‐FISH technique (Fig. 3H).

### RAP1 loss triggers DNA damage and accelerates the DDR onset in telomerase‐deficient mice

Critically short and uncapped telomeres are recognized as DNA double‐strand breaks and, as such, are bound by γH2AX (Hao *et al*., 2004). In turn, telomere damage elicits a DDR characterized by activation of p53 and the cell cycle inhibitor p21 that leads to cessation of cellular proliferation and cell death, ultimately causing tissue failure and various age‐related pathologies (Flores *et al*., 2005; Martinez & Blasco, 2010).

**Figure 4:**
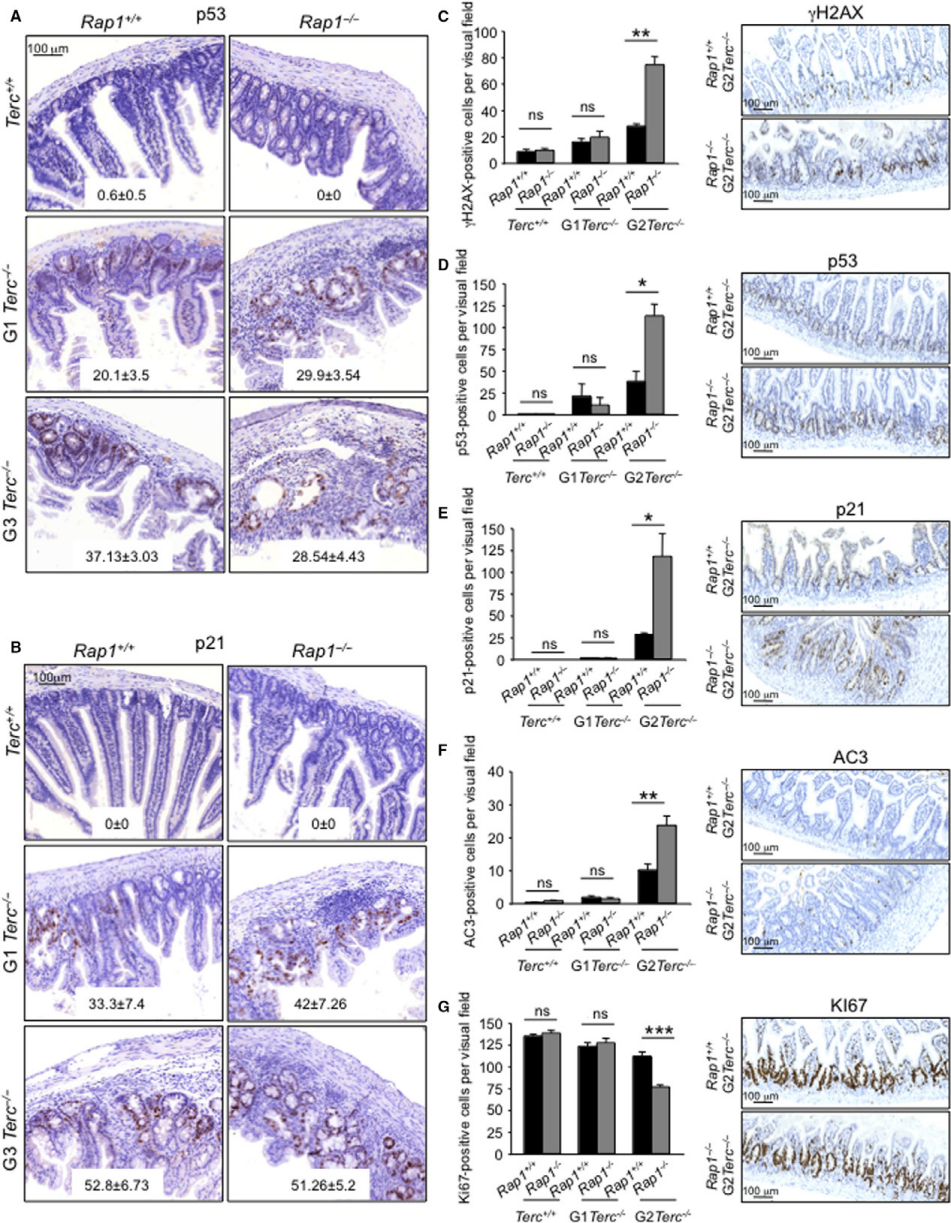
RAP1 loss enhances DNA damage and accelerates the DDR onset leading to a decrease in proliferation and increase in apoptosis in telomerase‐deficient mice. (A, B) Representative immunohistochemistry images of small intestine sections of mice at time of death of the indicated genotype stained for p53 (A) and p21(B). The mean number of p53‐ and p21‐positive cells per visual fields and standard error are shown within the images. The comparison between *Rap1* ^+/+^ and *Rap1* ^−/−^ from different *Terc* generations (G0‐G1‐G3) did not reach statistical significance by Student's *t*‐test. (C–G) Percentage of γH2Ax (C)‐, p53 (D)‐, p21 (E)‐, active caspase‐3 (F)‐, and KI67 (G)‐positive cells in small intestine sections of healthy young mice of the indicated genotype. Representative immunohistochemistry images are shown to the right en each case. Statistical significance was calculated by Student's *t*‐test analysis. (*n*) Five mice per each genotype. *p<0.05; **, *p*<0.01, ***p<0.001, ns: no significant.

We addressed whether the earlier onset of GI atrophy in G2‐*Rap1* ^−/−^ *Terc* ^−/−^ was associated with an earlier induction of DNA damage and its associated DDR. We quantify the number of γH2AX‐, p53‐, and p21‐positive cells in the intestine of mice of the different genotypes under study. At their time of death, we did not see significant differences in p53 and p21 levels in the GI tract between different *Rap1* ^+/+^ and *Rap1* ^−/−^ cohorts (Fig. 4A,B), possibly owing to the fact that these mice already had overt intestinal pathologies. Thus, we analyzed these markers at an earlier time in asymptomatic mice of 20–50 weeks of age (Fig. 4C–E). We did not find significant differences in the numbers of γH2AX‐, p53‐, and p21‐positive cells between *Rap1* ^+/+^ *Terc* ^+/+^ and *Rap1* ^−/−^ *Terc* ^+/+^. However, late‐generation G2‐*Rap1* ^−/−^ *Terc* ^+/+^ mice presented a higher DNA burden and increased p53 and p21 levels compared to G2‐*Rap1* ^+/+^ *Terc* ^+/+^ (Fig. 4C–E). Furthermore, G2‐*Rap1* ^−/−^ *Terc* ^−/−^ intestines showed higher number of apoptotic cells (positive for active caspase 3) and reduced proliferation (Ki67‐positive cells) compared to G2‐*Rap1* ^+/+^ *Terc* ^−/−^ (Fig. 4F,G).

Altogether, these results demonstrate that *Rap1* deletion in the context of telomerase deficiency anticipates the induction of p53 and p21 within tissues, concomitant with increased apoptosis and decreased proliferation rates in the GI track of late‐generation telomerase‐deficient mice, which in turn is accompanied by an anticipated onset of degenerative pathologies and earlier death.

### Increased RAP1 genomic occupancy with decreasing telomere length

Recruitment of RAP1 to telomeres occurs through interactions with TRF2, and therefore depended on the number of TRF2 molecules bound to telomeric DNA (Li *et al*., 2000; Arat & Griffith, 2012; Bandaria *et al*., 2016). Mammalian RAP1 also binds nontelomeric genomic sites where it regulates gene expression (Sarthy *et al*., 2009; Martinez *et al*., 2010, 2013; Yang *et al*., 2011; Yeung *et al*., 2013). We hypothesize here that telomere length could affect the ratio between telomere‐bound RAP1 and genomic DNA‐bound RAP1 that is available to perform its transcriptional regulatory function.

**Figure 5:**
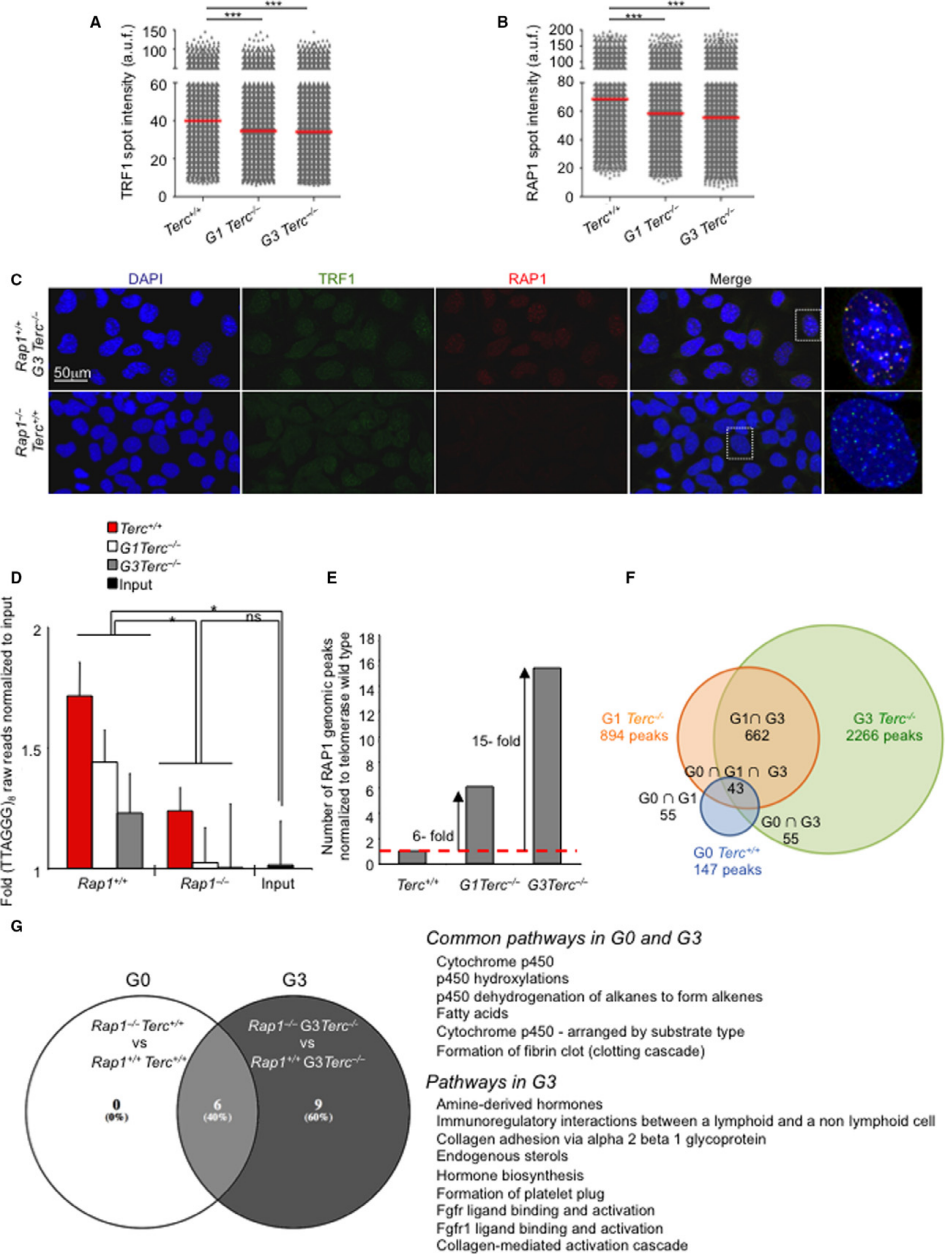
Progressive increase in extratelomeric RAP1‐binding sites in successive generations of telomerase‐deficient MEFS. (A, B) Quantification of TRF1 (A) and RAP1 (B) foci intensity in nuclei of *Rap1* ^+/+^ *Terc* ^+/+^ and in G1‐G3 *Rap1* ^+/+^ *Terc* ^−/−^ MEFs by immunofluorescence. *Rap1* ^−/−^ *Terc* ^+/+^ cells were used as negative controls. Co‐localization of RAP1 with TRF1 foci was used as control for telomere localization. The Student′s *t*‐test was used for statistical analysis. (*n*) Two independent MEFs per genotype. (C) Representative images of TRF1 (green foci) and RAP1 (red foci) doubly IF corresponding to G3 *Rap1* ^+/+^ *Terc* ^−/−^ and to *Rap1* ^−/−^ *Terc* ^+/+^ samples. (D) Fold change amount of 50‐bp raw ChIP‐seq reads (before genome alignment) containing perfect TTAGG_8_ repeats in *Rap1* ^+/+^ *Terc* ^+/+^ and (G1‐G3) *Rap1* ^+/+^ *Terc* ^−/−^ MEFs normalized to input sample. *Rap1* ^−/−^ *Terc* ^+/+^ and (G1‐G3) *Rap1* ^−/−^ *Terc* ^−/−^ MEFs were also analyzed as negative controls. The Student's *t*‐test was used for statistical analysis (E) Number of RAP1 genomic binding sites identified by ChIP‐seq analysis in *Rap1* ^+/+^ *Terc* ^+/+^ and (G1‐G3) *Rap1* ^+/+^ *Terc* ^−/−^ MEFs. The fold increases in G1 and G3 samples as compared to *Rap1* ^+/+^ *Terc* ^+/+^ are indicated. (F) Venn diagram showing the number of overlapping RAP1 genomic binding peaks among *Terc* ^+/+^ and G1‐G3 *Terc* ^−/−^ samples. The total number of genomic binding sites in each group is indicated. (G) Venn diagram showing the overlapping between the downregulated pathways in *Rap1* ^−/−^ *Terc* ^+/+^ and G3 *Rap1* ^−/−^ *Terc* ^−/−^ vs. their *Rap1* ^+/+^ counterparts according to GSEA analysis (FDR < 0.25). Note that those pathways found deregulated in G0 were also found downregulated in G3. In G3, cells lacking RAP1 showed additional downregulated pathways related to hormone biosynthesis, fibroblast growth factor receptor, and adhesion that were not observed in G0. *p<0.05; ***p<0.001, ns: no significant.

To address this, we first studied whether the total cellular RAP1 level varied with telomere shortening. To do this, we analyzed RAP1 protein levels by Western blot analysis with whole cell extract from immortalized *Rap1* ^+/+^ *Terc* ^+/+^, *Rap1* ^−/−^ *Terc* ^+/+^, G1 *Rap1* ^+/+^ *Terc* ^−/−^, G1 *Rap1* ^−/−^ *Terc* ^−/−^, G3 *Rap1* ^+/+^ *Terc* ^−/−^, and G3 *Rap1* ^−/−^ *Terc* ^−/−^ MEFs. We found that progressively shorter telomeres did not affect total RAP1 protein level (Fig. S3A, Supporting information). We then determined the RAP1 protein levels present at telomere foci by performing double immunofluorescence (IF) with RAP1 and TRF1 antibodies. We used *Rap1* ^−/−^ *Terc* ^+/+^ cells as negative control for RAP1 IF (Fig. 5A–C). We found a significant and progressive decrease in both TRF1 and RAP1 at telomere foci with decreasing telomere length in G1‐G3 telomerase‐deficient cells (Fig. 5A,B). These results suggest that telomere shortening owing to telomerase deficiency results in decreased RAP1 levels at telomere foci.

To determine whether the RAP1 genomic chromatin‐binding peaks also changed with telomere shortening, we performed a chromatin immunoprecipitation sequencing (ChIP‐seq) assay using the same immortalized MEFs (passage 10) used for the IF analysis (Fig 5A–C). The (G0‐G1‐G3) *Rap1*‐null MEFs were processed in parallel to validate the specificity of RAP1‐associated peaks obtained in *Rap1* WT MEFs. Over 22 million of raw 50‐bp reads were collected for each sample (Table S1, Supporting information). First, we analyzed the representation of the telomeric repeat TTAGGG in the ChIP‐seq raw reads of (G0‐G1‐G3) *Rap1* ^+/+^ vs. (G0‐G1‐G3) *Rap1* ^−/−^ samples before genome alignment. We found a strong overrepresentation of 50‐bp sequences containing the (TTAGGG)_8_ repeat in WT *Rap1* ChIP‐seq as compared to *Rap1*‐null controls, which showed similar levels to input DNA (Fig. 5D). In addition, we observed a progressively reduced number of telomeric reads in successive generations G1‐G3 *Rap1* ^+/+^ *Terc* ^−/−^ compared with *Rap1* ^+/+^ *Terc* ^+/+^ samples, thus confirming that the level of telomere‐bound RAP1 is decreased with telomere shortening, in agreement with the RAP1 IF data (Fig. 5B).

Identification of RAP1‐bound genomic binding sites revealed 147, 894 and 2,266 nontelomeric peaks in the comparison between the *Rap1* WT and the *Rap1*‐null ChIP‐seq G0, G1, and G3 datasets, respectively, at 10E‐03 *P*‐value cutoff (GEO accession number, GSE79996). Thus, the number of RAP1 genomic binding peaks was progressively increased in cells with progressively shorter telomeres, 6‐ and 15‐fold in G1 and G3 *Terc* ^−/−^ higher than in *Terc* ^+/+^ WT MEFs, respectively (Fig. 5E). In addition, approximately 40% of the G0 peaks were also found in G1 and G3, and 75% of the peaks identified in G1 were also detected in G3 (Fig. 5F). A total of 1,604 new RAP1 genomic biding sites were observed in G3 (Fig. 5F). Altogether, these results demonstrate that telomere length affects the ratio between telomere‐bound RAP1 and genomic DNA‐bound RAP1, leading to decreased RAP1 at telomeres and increased RAP1 occupancy at the chromosome arms.

We also confirmed our previous findings (Martinez *et al*., 2010) that the RAP1 peak density distribution in telomerase‐proficient MEFs was progressively decreasing along the chromosome arms toward the centromere, presenting the highest density at the subtelomeric region (Fig. S3B, Supporting information). Interestingly, this trend was inverted in G1 and G3 telomerase‐deficient MEFs that present a more evenly distributed peak density along the chromosome arms (Fig. S3B, Supporting information). These findings indicate that telomere shortening not only results in lower RAP1 binding at telomeres but also at subtelomeric sites.

### Effects of shortened telomeres on RAP1‐mediated transcription

To address whether the increased genomic RAP1 occupancy observed in *G1‐G3 Terc* ^−/−^ cells resulted in transcriptional changes, we carried out gene expression analysis by RNA sequencing (RNAseq) analysis from the same immortalized MEFs used for ChIP‐seq above. The results have been deposited in GEO (GEO accession number, GSE79996). Then, we performed GSEA analysis in G0‐G3 *Rap1* ^−/−^ MEFs (Tables S2 and S3, Supporting information, Fig. 5G). GSEA revealed significant downregulation of pathways involved in lipid metabolism in G0‐G3 *Rap1* ^−/−^ cells, being the number of downregulated pathways higher in G3 *Rap1* ^−/−^ than in G0 *Rap1* ^−/−^. Thus, in G3 *Rap1* ^−/−^ MEFs, pathways related to hormone biosynthesis, adhesion, and fibroblast growth factor receptor were downregulated as compared to RAP1‐proficient counterparts. These results are in agreement with the proposed role of RAP1 in the transcriptional activation of genes involved in fatty acid metabolism (Martinez *et al*., 2010) and with the increased numbers of RAP1‐genomic binding sites in G3 *Rap1* ^+/+^ as compared to *Rap1* ^+/+^ (Fig. 5E–G). Interestingly, GSEA analysis did not reveal any upregulated pathways in G3 *Rap1* ^−/−^ cells, while several pathways related to cell proliferation, extension of telomere, DNA repair, and carbohydrate–nucleid acid—amino acid metabolism were significantly upregulated in G0 *Rap1* ^−/−^ (Table S3, Supporting information). This observation likely reflects that *Rap1* deletion induces a basal state of telomere uncapping that is being repaired by different DNA damage repair pathways, that is, homologous recombination, ATM signaling, and GG‐NER. The fact that no differences in the activation of these pathways were detected between G3 *Rap1* ^+/+^ and G3 *Rap1* ^−/−^ may indicate that in the context of critically short telomeres (G3), both *Rap1* ^+/+^ and *Rap1* ^−/−^ MEFs present dysfunctional telomeres and therefore similarly activate these DNA damage repair‐related pathways. Interestingly, 0.44% of the genes whose expression was altered > 1.5 fold in G0 *Rap1* ^−/−^ presented a RAP1‐genomic binding peak while this figure was increased to 2.3% in G3 *Rap1* ^−/−^, consistent with a higher number of RAP1‐binding peaks G3 *Rap1* ^+/+^ than in G0 *Rap1* ^+/+^ MEFs (Fig. 5F).

To explore the biological pathways affected by *Rap1* deletion, we performed functional analysis with the significantly deregulated genes (FDR < 0.05) between G0‐G1‐G3 *Rap1* ^−/−^ cells compared to G0‐G1‐G3 *Rap1* ^+/+^ cells. For this, we used the Ingenuity software to find disorders and disease groups that were significantly affected (FDR < 0.05) in the three groups (Fig. S4, Supporting information). We found similar biological functions related with disease in the three groups. We did not detect deregulation of any particular disorder/disease in cells with shortened telomeres compared to telomerase‐proficient groups that could underline the reduced survival observed in G3 *Rap1* ^−/−^ mice. These results suggest that reduced survival in the *Rap1 Terc* doubly deficient mice is mainly due to RAP1 function in telomere homeostasis.

## Discussion

Here, we demonstrate that RAP1 exerts an important role in telomere maintenance and telomere protection in the context of telomerase deficiency. In line with this, mice lacking both RAP1 and telomerase show a progressive decreased survival with increasing mouse generations compared with telomerase single mutants.

**Figure 6:**
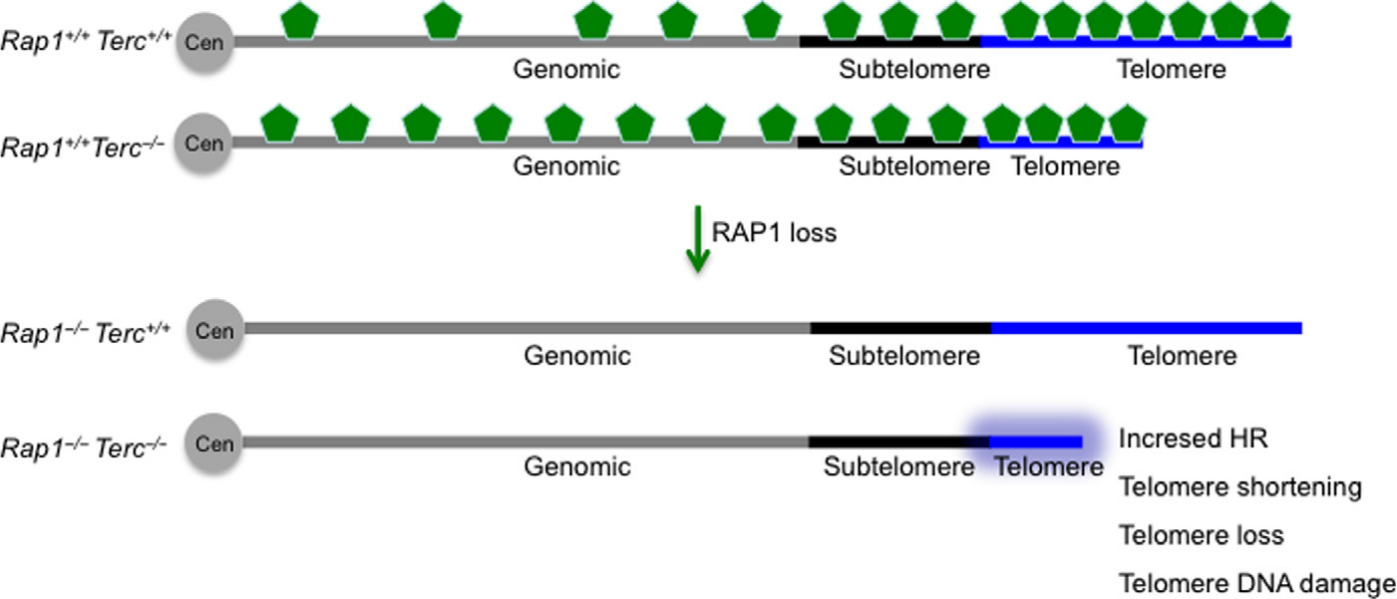
Proposed model for RAP1 role in telomere function. Cells with normal telomeres show an increased density of RAP1 peaks toward the subtelomeres and telomere, while this density decreases toward the centromere. As telomeres shorten associated with increasing generations of telomerase‐deficient mice, RAP1 occupancy at telomeres decreases while it increases at extratelomeric genomic sites. Furthermore, RAP1 enrichment at subtelomeric sites is lost when telomeres shortened. Interestingly, while RAP1 loss in cells with normal telomeres is tolerated and does not induce significant telomere shortening or induction of severe DNA damage signaling at telomeres, RAP1 deficiency in a context of telomerase deficiency and critically short telomeres augments telomere homologous recombination, further accelerating telomere shortening and chromosomal instability. We propose a previously unnoticed role for RAP1 in telomere maintenance in the context of telomerase deficiency and short telomeres, which in turn results in an anticipated appearance of degenerative capacities and shorter lifespan in mice.

In particular, we observe that telomere erosion due to telomerase deficiency is enhanced in the absence of RAP1, leading to an earlier onset of DNA damage and its consequent DDR (Fig. 6). As a consequence, we observe an accelerated onset and progression of degenerative pathologies (i.e. GI atrophies) in G3 *Rap1* ^−/−^ *Terc* ^−/−^ compared to G3 *Rap1* ^+/+^ *Terc* ^−/−^, thus leading to shorter mouse survival. These findings unveil a previously unnoticed role for RAP1 in telomere maintenance and tissue homeostasis. Importantly, we observed the most drastic pathological effects of RAP1 deficiency in the context of telomerase deficiency in proliferative tissues such as the intestinal tract, but not in the low‐proliferating tissues such as liver, lung, spleen, heart, or kidneys, suggesting that RAP1 is particularly important to maintain homeostasis of the highly proliferative tissues. In fact, RAP1‐mediated effects on telomere shortening are only observed in proliferative tissues such as intestines and blood indicating a cell‐/tissue‐specific RAP1 effect. The mechanisms underlying the accelerated telomere shortening could be explained by a faster cycling rate and/or increased ROS in *Rap1* ^−/−^ *Terc* ^−/−^ as compared to *Rap1* ^+/+^ *Terc* ^−/−^ intestinal cells. These results are in agreement with recent findings supporting that interaction between RAP1 and TRF2 is important for telomere function and chromosome‐end protection (Arat and Griffith 2012; Rai *et al*., 2016). In particular, interaction between RAP1 and TRF2 significantly increases the binding affinity of the complex for double (ds) telomeric repeats and for telomeric 3′ ends (Arat *et al*. 2012). In addition, the TRF2/RAP1 complex has higher ability to remodel telomeric DNA compared to either component alone. On long telomeres, the TRF2‐RAP1 complex can initiate t‐loop formation and prevention of the DDR. Similarly, on short telomeres where the t‐loop cannot be formed, the TRF2/RAP1 complex can still bind to the ds‐ss telomere junction due its high affinity for this structure and suppress the unnecessary DDR by blocking the end and making it less accessible to DNA repair machinery and thereby exerting a protective role (Arat *et al*. 2012). Thus, our results could be in agreement with a model in which t‐loop formation of critically short telomeres after replication is further compromised in RAP1‐depleted cells, being more susceptible to telomere degradation by nucleases. Indeed, RAP1 was recently reported to protect from end‐to‐end fusions in cells with topologically compromised telomeric DNA with significantly reduced cellular t‐loop content (Benarroch‐Popivker *et al*., 2016). In agreement with this, G3 *Rap1* ^−/−^ cells present higher incidence of end‐to‐end fusions (see Fig. 3F).

RAP1 has also been shown to be essential to protect telomeres from homology‐directed repair as its loss induces telomeric sister chromatin recombination (Martinez *et al*., 2010; Sfeir *et al*., 2010; Rai *et al*., 2016). Here, we confirm a moderate increase in telomeric homologous recombination (HR) in the absence of RAP1 regardless of the underlying telomere length, supporting an antirec function of RAP1. Indeed, it has recently been shown that RAP1 cooperates with the basic domain of TRF2 (TRF2^B^) to repress the HR factors PARP1 and SLX4 localization to telomeres. In the absence of RAP1 and TRF2^B^, PARP1 and SLX4 promote telomere resection that results in telomere loss and the generation of telomere‐free chromosome fusions mediated by the HR pathway (Rai *et al*., 2016). Thus, it is tempting to speculate that the enhanced telomere shortening observed in telomerase‐deficient cells lacking RAP1 is likely due to increased telomeric HR in the absence of RAP1 (Fig. 6). In addition, it is also possible that telomerase and RAP1 cooperate to protect telomeres from degradation during replication of telomeric DNA tracts through the antirec properties of RAP1.

We also found that RAP1 deficiency did not abolish the tumor suppressor role of short telomeres and telomerase deficiency (Greenberg *et al*., 1999; Gonzalez‐Suarez *et al*., 2000). This observation is particularly interesting in light of a previous report suggesting a tumor suppressor role for RAP1 via modulation of the NF‐κB‐mediated pathway (Teo *et al*., 2010). However, our results clearly show that RAP1 deficiency does not affect spontaneous cancer incidence throughout the lifespan of both telomerase‐deficient and telomerase WT mice (Martinez *et al*., 2013).

RAP1 protein does not contain a DNA‐binding domain (Li *et al*., 2000), and therefore, it is proposed to interact with yet unknown partners to accomplish its transcriptional function (Martinez *et al*., 2010). However, human RAP1 can bind *in vitro* to DNA with a preference for double‐strand–single‐strand junction in a sequence‐independent manner (Arat *et al*. 2012), thus raising the possibility that direct RAP1 binding to DNA could at least partially account for nontelomeric function at internal sites on the chromosomes. The recruitment of RAP1 to the telomeres is accomplished through interactions with the shelterin component TRF2 and is thereby dependent on the number of TRF2 molecules bound to the telomeric DNA repeats, which in turn is proportional to the length of telomeres (Li *et al*., 2000; Arat & Griffith, 2012; Bandaria *et al*., 2016). Telomere length is highly dynamic and does vary among different cell types and along aging (Harley *et al*., 1990; Liu *et al*., 2007; Flores *et al*., 2008). Thus, it is therefore conceivable that telomere length would affect the ratio between telomere‐bound RAP1 and genomic DNA‐bound RAP1 that is available to perform its transcriptional regulatory function. Indeed, in telomerase‐deficient senescent yeast, the levels of Rap1 at telomeres decrease concomitantly with an increase in Rap1 occupancy at extratelomeric sites already bound in nonsenescent cells as well as at other newly identified genomic sites denoted as new Rap1 targets at senescence (Platt *et al*., 2013). In addition, Rap1 delocalization contributes to the transcriptional changes observed in senescent cells and it was suggested that its release from telomere foci is a coordinated genomic response to telomere shortening (Platt *et al*., 2013). In agreement with this notion, here, we find that short telomeres owing to telomerase deficiency result in decreased RAP1 levels at telomeres as determined by whole‐genome ChIP sequencing, at the same time that increases RAP1 peaks at extratelomeric genomic sites in cells (Fig. 6). We also find that the known RAP1 enrichment at subtelomeric sites is lost as telomeres shorten with increasing mouse generations in the absence of telomerase, thus suggesting potential effects in subtelomeric gene silencing (Fig. 6). Accordingly, gene expression profiling in RAP1‐proficient and RAP1‐deficient G3‐*Terc* ^−/−^ MEFs revealed a moderate higher numbers of deregulated pathways in the absence of RAP1. These pathways were related to hormone biosynthesis, adhesion, and fibroblast growth factor receptor. Our data support that RAP1‐mediated effects on the survival of telomerase‐deficient mice are through its function in telomere homeostasis. However, given that transcriptional profiling in this work was performed in SV40‐LT immortalized MEFs, affected in many different pathways including p53 and RB, we cannot rule out that decreased survival in *Rap1* ^−/−^ *Terc* ^−/−^ compound mice could also partially be attributed to RAP1 role in transcriptional regulation. Future work aimed to analyze the RAP1‐mediated transcriptional effects in different tissue types of late‐generation telomerase‐deficient mice is needed to ultimately unveil RAP1 role in the survival of telomerase‐deficient mice.

The deleterious *in vivo* effects of single *Rap1* deletion are manifested in the liver and white fat tissues resulting in obesity and hepatic steatosis (Martinez *et al*., 2013; Yeung *et al*., 2013). However, telomerase deficiency in combination with RAP1 depletion abrogates the obese phenotype and liver pathologies observed in *Rap1* single knockout mice. One likely explanation to this observation is that the severe intestinal degenerative pathologies induced by telomerase deficiency impede proper nutrient absorption at the GI track, thereby inhibiting lipid accumulation. Alternatively, few critically short telomeres in liver cells as a consequence of telomerase deficiency could trigger a metabolic program counteracting the one induced by RAP1 loss.

Our findings demonstrate that although RAP1 is not a key factor in telomere capping under conditions of a sufficient telomere reserve, in the settings of telomerase deficiency, RAP1 exerts an important function in telomere protection and telomere length maintenance, which in turn is important to delay the appearance of degenerative diseases and for organismal longevity. These previously uncovered findings explain its evolutionary conservation as a shelterin component in mammalian cells.

## Experimental procedures

### Generation of *Rap1* Terc double knockout mice

*Rap1Terc* knockout mice, Rap1^−/−^Terc^−/−^, were generated by crossing *Rap1* ^−/−^ (C57BL6/129SV; 90%: 10%) (Martinez *et al*., 2013) with *Terc* ^−/−^ mice (C57BL6; 100%) (Blasco *et al*., 1997). The resulting double heterozygous breeding pairs were used to generate G1 of single *Terc* ^−/−^ and of double *Rap1* ^−/−^ *Terc* ^−/−^. Mouse colonies of successive generations, G2 and G3, were generated by intercrossing G1 and G2 *Rap1* ^+/−^ *Terc* ^−/−^ pairs, respectively. All mice were generated and maintained at Animal Facility of the Spanish National Cancer Research Centre (CNIO) under specific pathogen‐free conditions in accordance with the recommendation of the Federation of European Laboratory Animal Science Associations.

## Author contributions

MAB and PM designed experiments and wrote the manuscript. PM performed the experiments. GG‐L and DP performed de RNAseq and ChIPseq analysis. JMF performed the pathology analyses.

## Funding

Research in the Blasco laboratory is funded by Spanish Ministry of Economy and Competitiveness (MINECO and FEDER) Project RETOS (SAF2013‐45111‐R), the European Research Council (ERC) Project TEL STEM CELL (ERC‐2008‐AdG/232854), and Fundación Botín.

## Conflict of interest

None declared.

## Supporting information


**Fig. S1** Kaplan–Meyer survival curves (A,C) and (B,D) Median survival from Kaplan–Meyer plots of *Rap1* ^+/+^ *Terc* ^+/+^, *Rap1* ^−/−^ *Terc* ^+/+^, G1 *Rap1* ^+/+^ *Terc* ^−/−^, G1 *Rap1* ^−/−^ *Terc* ^−/−^, G2 *Rap1* ^+/+^ *Terc* ^−/−^, G2 *Rap1* ^−/−^ *Terc* ^−/−^, G3 *Rap1* ^+/+^ *Terc* ^−/−^ and G3 *Rap1* ^−/−^ *Terc* ^−/−^ males (A,B) and females (C,D).
**Fig. S2** (A) Incidence of kidney, lung, spleen and heart pathologies at death point of mice of the indicated genotypes.
**Fig. S3** (A) Quantification of total cellular RAP1 levels in cell extract of two independent immortalized MEFS of the indicated genotypes by Western blot (WB).
**Fig. S4** Disorders and diseases found transcriptionally deregulated (FDR < 0.05) in (G0‐G1‐G3) RAP1‐deficient MEFs as compared tp RAP1‐proficient counterparts analyzed by Ingenuity software.

**Appendix S1** Experimental procedures.
**Table S1** Overall reads of 50 bases length obtained in Illumina ChIP‐seq.
**Table S2** Downregulated pathways in *Rap1* ^−/−^ *Terc* ^+/+^ vs. *Rap1* ^+/+^ *Terc* ^+/+^.
**Table S3** Upregulated pathways in *Rap1* ^−/−^ *Terc* ^+/+^ vs. *Rap1* ^+/+^ *Terc* ^+/+^.
