# Supplementary figures and images for: A genetic interaction between RAP1 and telomerase reveals an unanticipated role for RAP1 in telomere maintenance
Source: Aging Cell. 2016 Sep 1;15(6):1113–25. doi: 10.1111/acel.12517 (PMC5114719; doi:10.1111/acel.12517)

Fig. S1

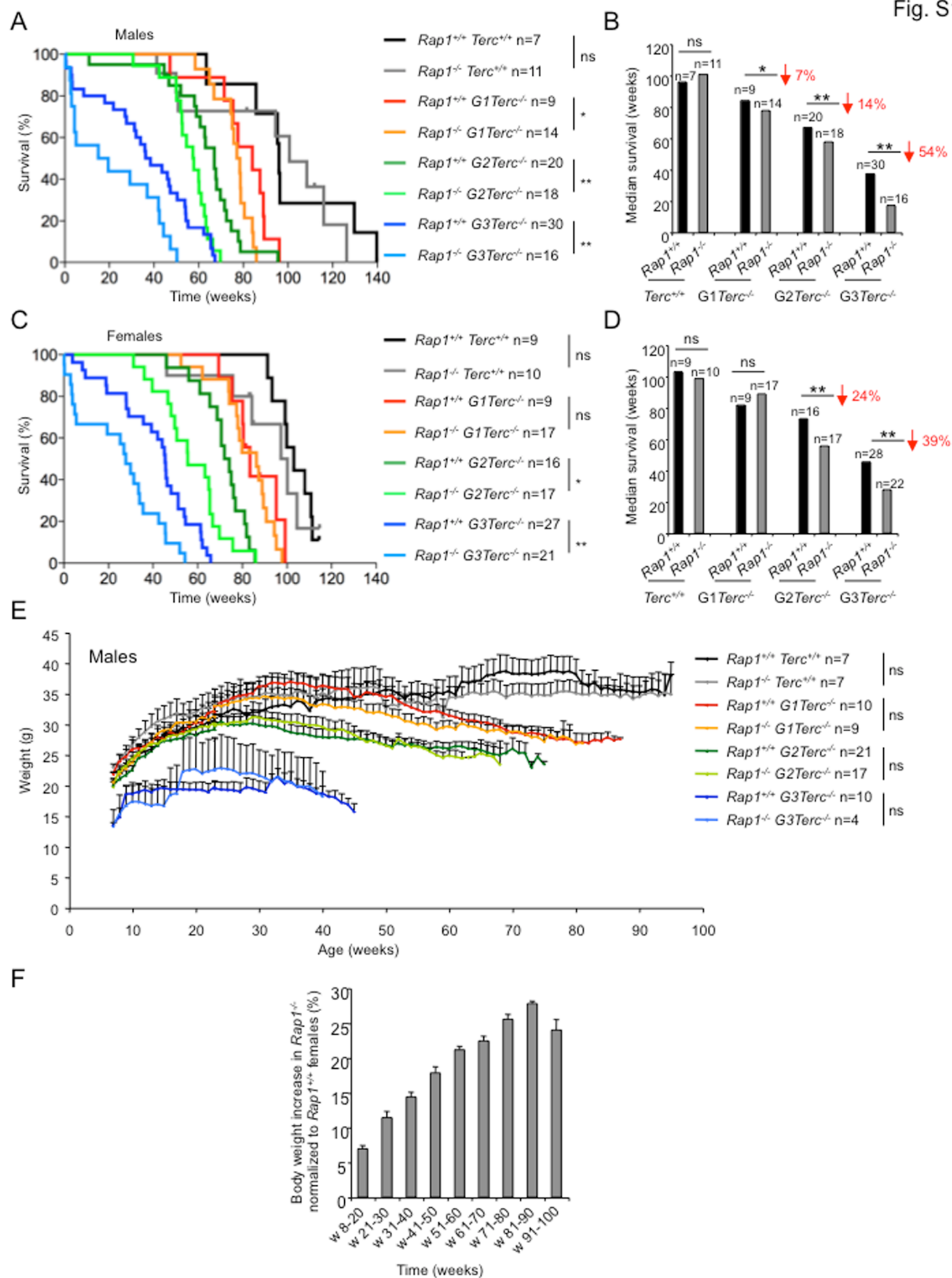

Fig. S2

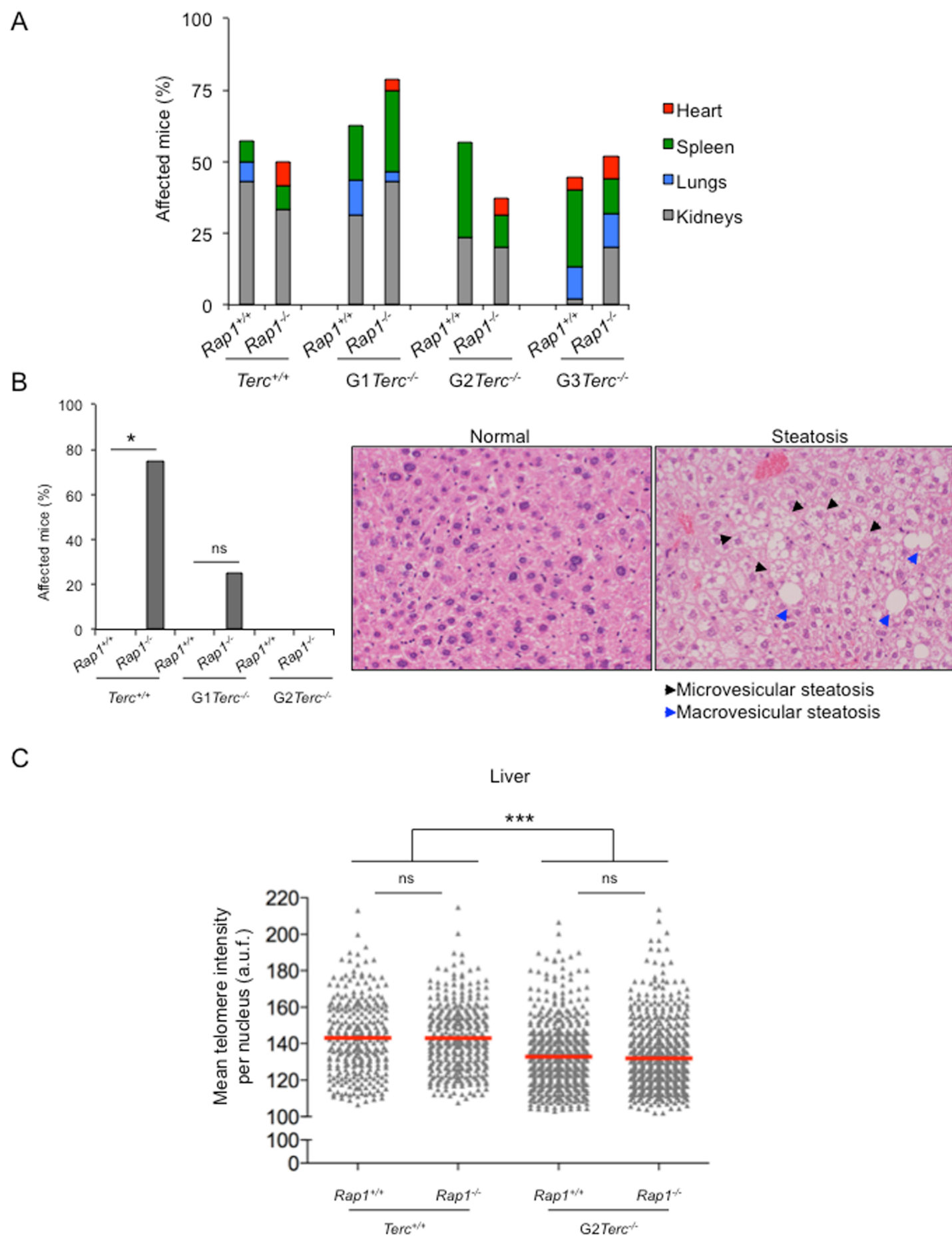

Fig. S3

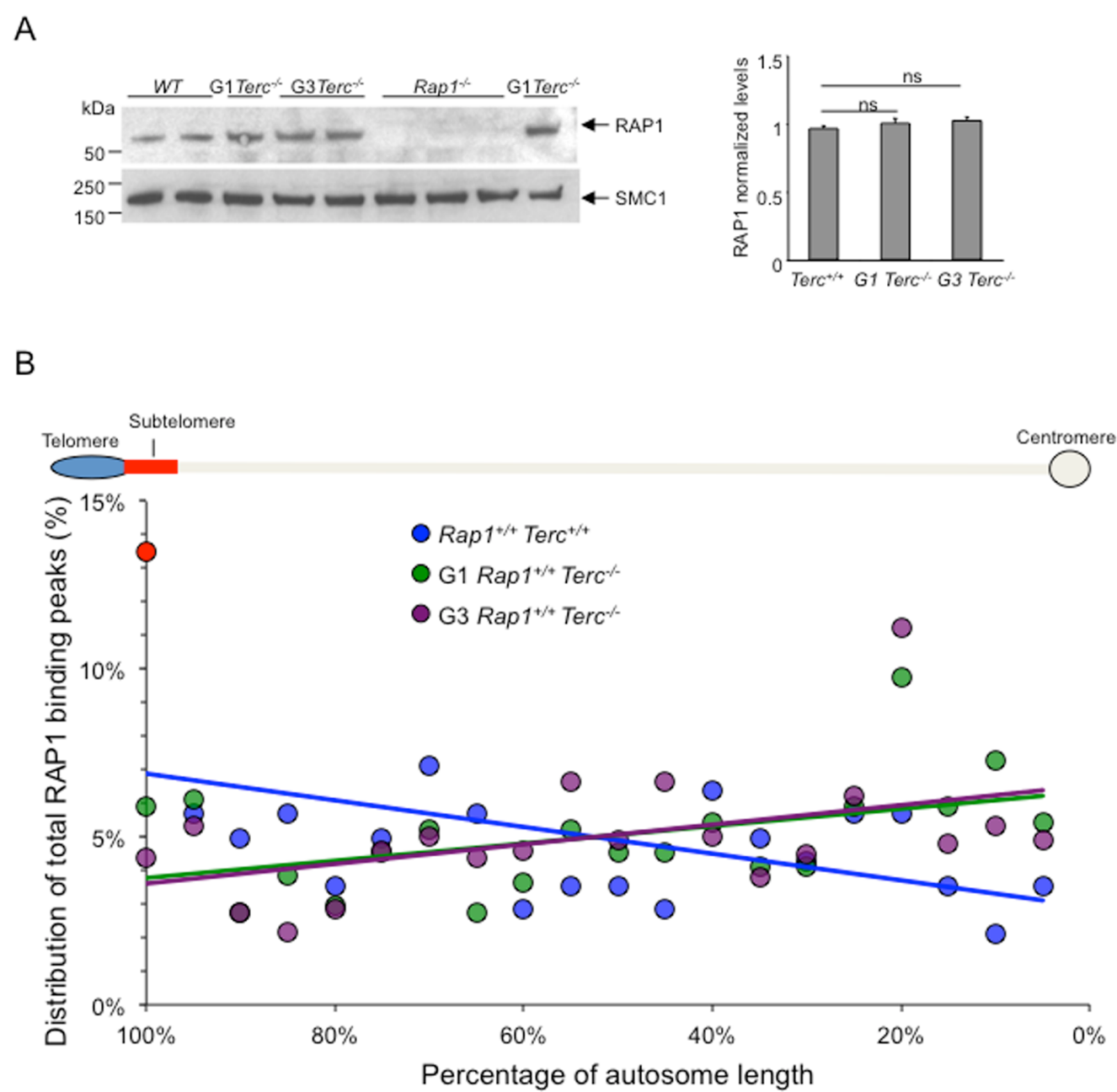

Fig S4.

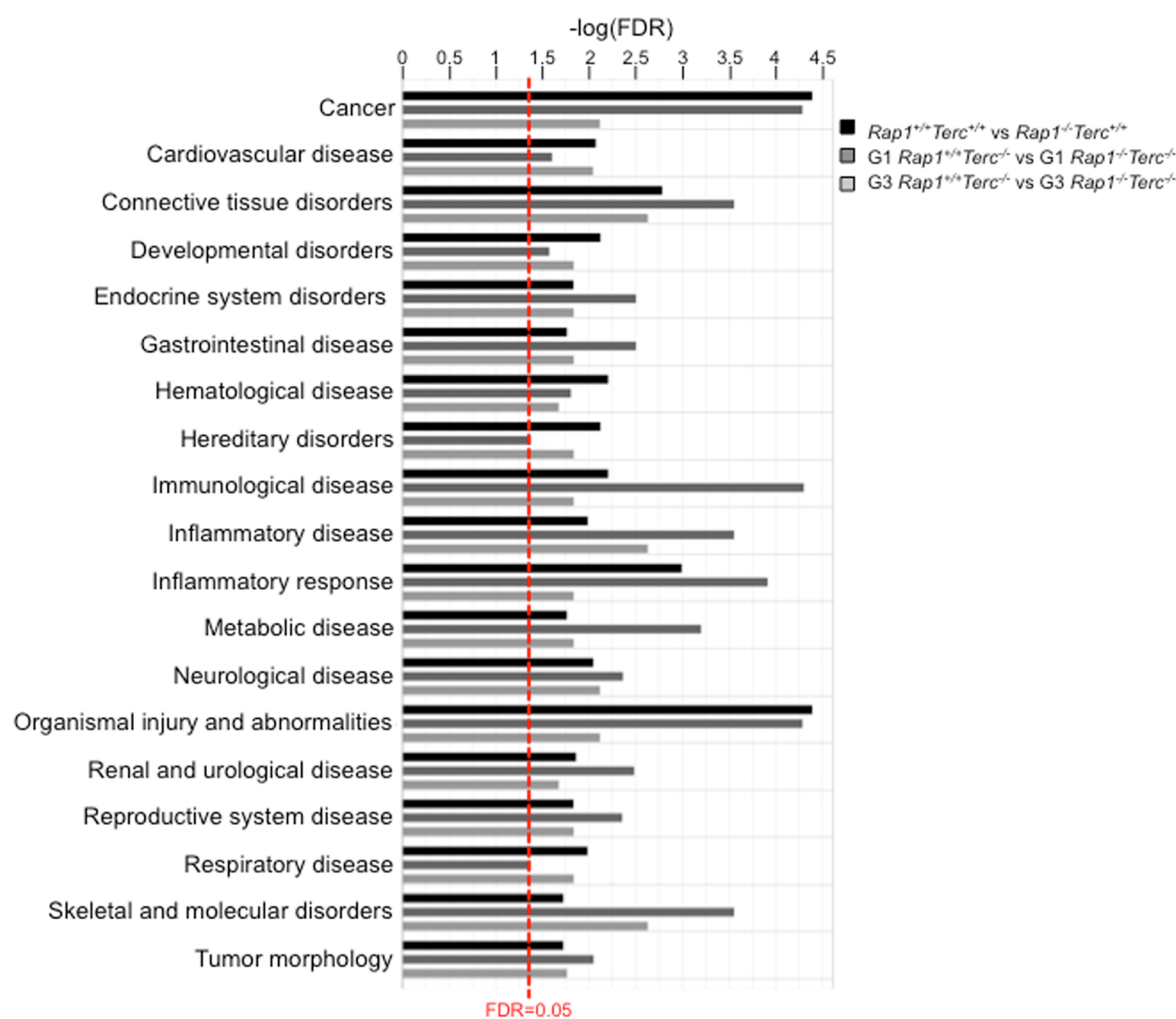

Supplement: Supplementary file 1 — Fig. S1 Kaplan–Meyer survival curves (A,C) and (B,D) Median survival from Kaplan–Meyer plots of Rap1 +/+ Terc +/+, Rap1 −/− Terc +/+, G1 Rap1 +/+ Terc −/−, G1 Rap1 −/− Terc −/−, G2 Rap1 +/+ Terc −/−, G2 Rap1 −/− Terc −/−, G3 Rap1 +/+ Terc −/− and G3 Rap1 −/− Terc −/− males (A,B) and females (C,D). Fig. S2 (A) Incidence of kidney, lung, spleen and heart pathologies at death point of mice of the indicated genotypes. Fig. S3 (A) Quantification of total cellular RAP1 levels in cell extract of two independent immortalized MEFS of the indicated genotypes by Western blot (WB). Fig. S4 Disorders and diseases found transcriptionally deregulated (FDR < 0.05) in (G0‐G1‐G3) RAP1‐deficient MEFs as compared tp RAP1‐proficient counterparts analyzed by Ingenuity software. [file ACEL-15-1113-s001.pdf]
