# Supplementary material for: A genetic interaction between RAP1 and telomerase reveals an unanticipated role for RAP1 in telomere maintenance
Source: Aging Cell. 2016 Sep 1;15(6):1113–25. doi: 10.1111/acel.12517 (PMC5114719; doi:10.1111/acel.12517)
Supplement: Supplementary file 2 — Appendix S1 Experimental procedures. Table S1 Overall reads of 50 bases length obtained in Illumina ChIP‐seq. Table S2 Downregulated pathways in Rap1 −/− Terc +/+ vs. Rap1 +/+ Terc +/+. Table S3 Upregulated pathways in Rap1 −/− Terc +/+ vs. Rap1 +/+ Terc +/+. [file ACEL-15-1113-s002.docx]

**Supporting Information**

**Experimental procedures**

**Telomere length quantitative fluorescence (Q-Fish) analyses on tissue sections and on metaphases**

Two independent immortalized MEFS per each genotype were incubated with 0.1µg/ml colcemide (Gibco) for 4 h at 37ºC and then fixed in methanol:acetic acid (3:1). Quantitative telomere fluorescence in situ hybridization (Q-FISH) was performed as described (Samper *et al.* 2000). Q-FISH directly on tissue sections was performed as previously described (Martinez *et al.* 2013). Confocal microscopy was performed at room temperature with a laser-scanning microscope (TSC SP5) using a Plan Apo 63Å-1.40 NA oil immersion objective (HCX). Maximal projection of z-stack images generated using advanced fluorescence software (LAS) were analyzed with Definiens XD software package. The DAPI images were used to detect telomeric signals inside each nuclei.

**Telomere recombination measurements using chromosome orientation FISH (CO-FISH)**

Exponentially growing primary MEFs were sub-cultured in the presence of 5’-bromo-2’-deoxyuridine (BrdU; Sigma) at a final concentration of 1x10^-5^ M, and then allowed to replicate their DNA once at 37ºC for 24 hours. Colcemide was added at a concentration of 0.1 µg/ml during the last 4 hours. Cells were then recovered and metaphases prepared as described (Samper *et al.* 2000). CO-FISH was performed as described (Bailey *et al.* 2004).

**Western blots**

Whole-cell extracts were prepared from immortalized MEFs of the indicated genotype and 50 μg of each extract were separated in 4-20% gradient SDS-polyacrilamide gels by electrophoresis. After transfer, the membranes were incubated with an anti-RAP1 (A300-306A from Bethyl laboratory) and anti-SMC1 (A300-055A from Bethyl laboratory). Antibody binding was detected after incubation with a secondary antibody coupled to horseradish peroxidase using enhanced chemiluminescence.

**Histopathology and immunohistochemistry**

Tissues were fixed in 10% buffered formalin, embedded in paraffin wax and sectioned at 5 mm. For pathological examination sections were stained with hematoxylin and eosin, according to standard procedures.

Liver pathologies were classified as either severe multifocal hepatitis or as non-alcoholic steatohepatitis (NASH). Hepatitis is characterized as lobular inflammation with lymphocytes, macrophages and neutrophiles infiltrates and hepatocellular ballooning degeneration. NASH is a progressive process that begins with lipid deposition in the liver, showing both macro and microvesicular fatty changes in hepatocyte’s cytoplasm followed by hepatocellular ballooning degeneration and lobular multifocal inflammation.

Intestinal atrophies found at death were classified as mild, medium or severe according to the pathological findings. Mild intestinal lesions are characterized by multifocal epithelia and glandular atrophy, showing architectural alterations of intestinal crypts as well as by the presence of diffuse inflammatory reaction with lymphocytes, macrophages and neutrophyles in lamina propria and submucosa. Medium intestinal lesions show multifocal areas in the mucosa lacking glands, the presence of degenerative epithelial and glandular cystic hyperplasia, the presence of diffuse inflammatory reaction in lamina propria and submucosa as well as hyperplasic reaction of gut associated lymphoid tissue (GALT). Severe intestinal atrophy is characterized by marked diffuse trasmural inflammation, regenerative epithelial and glandular hyperplasia that narrows the intestinal lumen, presence of cellular atypia in regenerated glands, presence of severe glandular atrophy and ulcers in the epithelium that increase the immflamatory response as well as by the abundance of hyperplasic reaction of Peyer’s patches.

Intestinal and immflamatory pathologies found in young mice at time point within their health span during which animals remain healthy with no pathological signs were classified as mild, medium or severe according to the pathological findings. Mild intestinal atrophy is characterized by multifocal mild shortening of the villi top in small intestine or in the folds in large intestine with no inflammatory reaction or architectural pathological glandular changes. Medium intestinal atrophy is characterized by generalizad moderate shortening of the villi top in small intestine and in the folds in large intestine, atrophy of glandular Paneth cells and a multifocal moderate subacute inflammation in lamina propria. Severe intestinal atrophy is characterized by generalized shortening or loss of the villi in small intestine and in the folds in large intestine, epithelial and glandular cystic hiperplasia and diffuse severe mixed inflammation in lamina propria.

Immunohistochemistry was performed on de-paraffinated intestine sections processed with 10 mM sodium citrate (pH 6.5) cooked under pressure for 2 min. Slides were washed in water, then in Buffer TBS Tween20 0.5 %, blocked with peroxidase, washed with TBS Tween20 0.5 % again and blocked with fetal bovine serum followed by another wash. The slides were incubated with the primary antibodies: mouse monoclonal to phospho-Histone H2AX (ser139) (JBW301, Millipore), rabbit monoclonal to Ki-67 antibody (SP6, Master Diagnostica), rabbit polyclonal to C3 cleaved-caspase 3 (Asp175) (Cell Signaling), rat monoclonal to p53 (POE316 A/E9, Monoclonal antibody core unit, CNIO) or rat monoclonal to p21 (291H/B5, Monoclonal antibody core unit, CNIO). Slides were then incubated with secondary antibodies conjugated with peroxidase from DAKO. Sections were lightly counterstained with hematoxylin and analyzed by light microscopy.

**Immunofluorescence staning techniques**

Immortalized MEFs were treated for 5 min with Triton-100 buffer (Munoz *et al.* 2009) for nuclear extraction, fixed 10 min in 4% buffered formaldehyde, permeabilized with 0.2% PBS-Triton for 10 min and blocked with 2% fetal bovine serum in PBS for 1h. Sampels were incubated O/N at 4ºC with a polyclonal rabbit anti-RAP1 (A300-306A from Bethyl laboratory) and with a rat monoclonal anti-TRF1 (Monoclonal antibody core facility, CNIO) antibody at 1:200 dilution. Slides were further incubated with 488-Alexa or 555-Alexa labeled secondary antibodies. Slides were mounted in Vectashield with 4',6-diamino-2-phenylindole (DAPI). Confocal microscopy was performed at room temperature with a laser-scanning microscope (TCS SP5; Leica) using a Plan Apo 63Å-1.40 NA oil immersion objective (HCX; Leica). Maximal projection of Z-stack images generated using advanced fluorescence software (LAS) was analyzed with the Definiens XD software package.

**ChIP-sequencing analyses and identification of RAP1-binding peaks**

Chromatin immunoprecipitation was performed as described (Martinez *et al.* 2010) using a rabbit polyclonal antibody against RAP1 (A300-306A from Bethyl laboratory). Biological duplicates of ChIP samples were independently processed into sequencing libraries. Input samples from both Rap1-null and WT MEFs were pooled and treated as a single input. 50-250bp fractions were processed through subsequent enzymatic treatments of end-repair, dA-tailing, and ligation to adapters as in Illumina's "TruSeq DNA Sample Preparation Guide" (part # 15005180 Rev. C). Libraries were applied to an Illumina flow cell for cluster generation (TruSeq cluster generation kit v5) and sequenced on an Illumina HiSeq2000 instrument by following manufacturer's protocols. Only high quality reads (Q>30) were used.

Genome alignment was performed with BWA 0.6.1 versus the mouse assembly MGSCv37 (NCBI Build 37) under default settings. All analysis pipelines were run using RUbioSeq v3.6.1 (Rubio-Camarillo *et al.* 2013). The experimental settings, sequences and analysis protocols of the ChIP-seq experiment have been deposited in GEO under the accession number GSE79996. Identification of RAP1-bound genomic binding sites was performed as follows. Sequenced reads from both biological replicates from each genotype and from (G0-G1-G3) *Rap1^+/+^* input samples (Support information Table 1) were pooled and aligned towards mouse genome referente (MGSCv37). The *Rap1^-/-^* samples in each generation (G0-G1-G3) were used as negative controls for the Rap1*^+/+^* samples in the peak calling step, using MACS v 2.0.10.20130712 (Zhang *et al.* 2008). Briefly, uniquely aligned 50 bp-length reads obtained in two independent runs were pooled into 3 paired datasets, corresponding to the Rap1 WT MEFs and Rap1-null MEFs (KO) in G0, G1 and G3 respectively. Genomic peaks obtained from input samples, when compared to a random background, were subtracted from the peaks lists to eliminate unspecific binding peaks. Only the reads having a unique alignment in the reference genome where used for the RAP1 binding sites determination, thus excluding reads consisting only of telomeric repeats. After format conversion and file cleaning, MACS2 software recommendations to analyze two-sample ChIP-seq experimental designs was applied using the WT reads as sample set and the KO reads as negative control in each condition. For each analysis, a random sample of high quality peaks is used to look for bimodal tag distributions, and then estimate the distance between the tags mapping to the Watson and the Crick strand, respectively. All reads are shifted by half that distance to model the true binding site. A dynamic local Poisson distribution model is then used to determine regions were the sample reads are significantly enriched which respect to the negative control reads. Those peaks passing a 10E-03 p-value threshold were used to determine the predicted RAP1 binding regions. A post-processing cleaning step was included to remove all regions enriched in the pooled input sample.

**Gene expression analysis by RNAseq**

Total RNA samples with RNA Integrity Numbers in the range 8.2-9.9 (Agilent 2100 Bioanalyzer) were used. PolyA+ fractions were purified and randomly fragmented, converted to double stranded cDNA and processed as in Illumina's "TruSeq Stranded mRNA Sample Preparation Part # 15031047 Rev. D" kit. The resulting directional cDNA libraries were sequenced as above.

Reads were aligned to the mouse genome (GRCm38/mm10) with Nextpresso (Grana *et al.* 2016), using TopHat-2.0.10, Bowtie 1.0.0 and Samtools 0.1.19.0; allowing two mis-matches and five multihits. Transcripts assembly, estimation of their abundances and differential expression were calculated with Cufflinks 2.2.1, using the mouse genome annotation data set GRCm38/mm10 from the UCSC Genome Browser.

GSEAPreranked was used to perform a gene set enrichment analysis of Reactome pathways. We used the RNA-seq gene list ranked by statistic, setting ‘gene set’ as the permutation method and we run it with 1000 permutations. We considered only those gene sets with significant enrichment levels (FDR q-value<0.25). Functional analyses of the differentially expressed gene lists (FDR<0.05) were carried out using Ingenuity Pathway Analysis software (Ingenuity Systems).

Statistical analysis

A log rank test was used to calculate statistical differences in median survival of the different mouse cohorts. A t-student test was used to calculate the statistical significance of the observed differences in median survival, chromosomal aberrations, telomeric sister chromatid exchange (T-SCE), γH2AX, p21, p53, AC3 and KI67. The Student t-test was used for statistical comparisons of the mean telomere length in MEFs and in tissue sections. A chi-square test was used to calculate statistical differences in pathologies.

**Accesion numbers**

The RNAseq and ChIPseq datasets have been deposited in the GEO database (GSE79996).

**References**

Bailey SM, Brenneman MA , Goodwin EH (2004). Frequent recombination in telomeric DNA may extend the proliferative life of telomerase-negative cells. *Nucleic Acids Res*. 32, 3743-3751.

Grana O, Rubio-camarillo M, Fernandez-Riverola F, Pisano DG , Gonzalez-Pena D (2016). Nextpresso: next generation sequencing expression analysis pipeline. *Current Bioinformatics*.

Martinez P, Gomez-Lopez G, Garcia F, Mercken E, Mitchell S, Flores JM, de Cabo R , Blasco MA (2013). RAP1 protects from obesity through its extratelomeric role regulating gene expression. *Cell Rep*. 3, 2059-2074.

Martinez P, Thanasoula M, Carlos AR, Gomez-Lopez G, Tejera AM, Schoeftner S, Dominguez O, Pisano DG, Tarsounas M , Blasco MA (2010). Mammalian Rap1 controls telomere function and gene expression through binding to telomeric and extratelomeric sites. *Nat Cell Biol*. 12, 768-780.

Munoz P, Blanco R, de Carcer G, Schoeftner S, Benetti R, Flores JM, Malumbres M , Blasco MA (2009). TRF1 controls telomere length and mitotic fidelity in epithelial homeostasis. *Mol Cell Biol*. 29, 1608-1625.

Rubio-Camarillo M, Gomez-Lopez G, Fernandez JM, Valencia A , Pisano DG (2013). RUbioSeq: a suite of parallelized pipelines to automate exome variation and bisulfite-seq analyses. *Bioinformatics*. 29, 1687-1689.

Samper E, Goytisolo FA, Slijepcevic P, van Buul PP , Blasco MA (2000). Mammalian Ku86 protein prevents telomeric fusions independently of the length of TTAGGG repeats and the G-strand overhang. *EMBO Rep*. 1, 244-252.

Zhang Y, Liu T, Meyer CA, Eeckhoute J, Johnson DS, Bernstein BE, Nusbaum C, Myers RM, Brown M, Li W , Liu XS (2008). Model-based analysis of ChIP-Seq (MACS). *Genome Biol*. 9, R137.

**Supplementary Tables**

**Supplementary Table 1:** Overall reads of 50 bases length obtained in Illumina ChIP-seq. Two independent biological replicates per genotype were processed, along a pool from al the six different (G0-G1-G2) *Rap1^+/+^* input samples. Alignments versus the MGSCv37 (NCBI Build 37) mouse genome assembly were made with BWA 0.6.1 software.

**Supplementary Table 2.**

Downregulated pathways in *Rap1^-/-^ Terc^+/+^* vs *Rap1^+/+^ Terc^+/+^*

| NAME | SOURCE | GENESET SIZE | FDR q-val |
| --- | --- | --- | --- |
| CYTOCHROME P450 | REACTOME | 26 | 0.030 |
| P450 HYDROXYLATIONS | REACTOME | 18 | 0.074 |
| P450 DEHYDROGENATION OF ALKANES TO FORM ALKENES | REACTOME | 19 | 0.083 |
| FATTY ACIDS | REACTOME | 15 | 0.140 |
| CYTOCHROME P450 - ARRANGED BY SUBSTRATE TYPE | REACTOME | 52 | 0.156 |
| FORMATION OF FIBRIN CLOT (CLOTTING CASCADE) | REACTOME | 30 | 0.193 |

Downregulated pathways in G3 *Rap1^-/-^Terc^-/-^* vs G3 *Rap1^+/+^Terc^-/-^*

| NAME | SOURCE | GENESET SIZE | FDR q-val |
| --- | --- | --- | --- |
| P450 HYDROXYLATIONS | REACTOME | 18 | 0.005 |
| CYTOCHROME P450 | REACTOME | 26 | 0.005 |
| P450 DEHYDROGENATION OF ALKANES TO FORM ALKENES | REACTOME | 19 | 0.006 |
| AMINE-DERIVED HORMONES | REACTOME | 16 | 0.005 |
| FATTY ACIDS | REACTOME | 15 | 0.004 |
| CYTOCHROME P450 - ARRANGED BY SUBSTRATE TYPE | REACTOME | 52 | 0.018 |
| FORMATION OF FIBRIN CLOT (CLOTTING CASCADE) | REACTOME | 30 | 0.036 |
| IMMUNOREGULATORY INTERACTIONS BETWEEN A LYMPHOID AND A NON-LYMPHOID CELL | REACTOME | 47 | 0.050 |
| RCOLLAGEN ADHESION VIA ALPHA 2 BETA 1 GLYCOPROTEIN | REACTOME | 16 | 0.061 |
| ENDOGENOUS STEROLS | REACTOME | 18 | 0.143 |
| HORMONE BIOSYNTHESIS | REACTOME | 78 | 0.160 |
| FORMATION OF PLATELET PLUG | REACTOME | 119 | 0.1625 |
| FGFR LIGAND BINDING AND ACTIVATION | REACTOME | 23 | 0.221 |
| FGFR1 LIGAND BINDING AND ACTIVATION | REACTOME | 20 | 0.212 |
| COLLAGEN-MEDIATED ACTIVATION CASCADE | REACTOME | 17 | 0.217 |

**Supplementary Table 3.** Upregulated pathways in *Rap1^-/-^Terc^+/+^* vs *Rap1^+/+^Terc^+/+^*.

| NAME | SOURCE | GENESET SIZE | FDR q-val |
| --- | --- | --- | --- |
| FORMATION OF A POOL OF FREE 40S SUBUNITS | REACTOME | 94 | 0 |
| EUKARYOTIC TRANSLATION TERMINATION | REACTOME | 85 | 0 |
| PEPTIDE CHAIN ELONGATION | REACTOME | 86 | 0 |
| ELECTRON TRANSPORT CHAIN | REACTOME | 62 | 0 |
| EUKARYOTIC TRANSLATION ELONGATION | REACTOME | 89 | 0 |
| ACTIVATION OF APC-C AND APC-C:CDC20 MEDIATED DEGRADATION OF MITOTIC PROTEINS | REACTOME | 78 | 0 |
| L13A-MEDIATED TRANSLATIONAL SILENCING OF CERULOPLASMIN EXPRESSION | REACTOME | 106 | 0 |
| 3 -UTR-MEDIATED TRANSLATIONAL REGULATION | REACTOME | 106 | 0 |
| APC-C-MEDIATED DEGRADATION OF CELL CYCLE PROTEINS | REACTOME | 85 | 0 |
| CAP-DEPENDENT TRANSLATION INITIATION | REACTOME | 113 | 0 |
| AUTODEGRADATION OF CDH1 BY CDH1:APC-C | REACTOME | 64 | 0 |
| APC-C:CDC20 MEDIATED DEGRADATION OF MITOTIC PROTEINS | REACTOME | 75 | 0 |
| CDC20:PHOSPHO-APC-C MEDIATED DEGRADATION OF CYCLIN A | REACTOME | 69 | 0 |
| EUKARYOTIC TRANSLATION INITIATION | REACTOME | 113 | 0 |
| APC-C:CDH1 MEDIATED DEGRADATION OF CDC20 AND OTHER APC-C:CDH1 TARGETED PROTEINS IN LATE MITOSIS-EARLY G1 | REACTOME | 72 | 0 |
| M-G1 TRANSITION | REACTOME | 56 | 0 |
| GTP HYDROLYSIS AND JOINING OF THE 60S RIBOSOMAL SUBUNIT | REACTOME | 106 | 0 |
| DNA REPLICATION PRE-INITIATION | REACTOME | 70 | 0 |
| ASSEMBLY OF THE PRE-REPLICATIVE COMPLEX | REACTOME | 56 | 0 |
| APC-C:CDC20 MEDIATED DEGRADATION OF SECURIN | REACTOME | 65 | 6.00E-03 |
| CDT1 ASSOCIATION WITH THE CDC6:ORC:ORIGIN COMPLEX | REACTOME | 50 | 1.40E-02 |
| ORNITHINE AND PROLINE METABOLISM | REACTOME | 59 | 4.00E-03 |
| CDK-MEDIATED PHOSPHORYLATION AND REMOVAL OF CDC6 | REACTOME | 49 | 5.00E-03 |
| ORNITHINE METABOLISM | REACTOME | 54 | 7.00E-03 |
| DNA REPLICATION | REACTOME | 91 | 9.90E-02 |
| METABOLISM OF AMINO ACIDS | REACTOME | 172 | 0.001 |
| FORMATION OF THE TERNARY COMPLEX, AND SUBSEQUENTLY, THE 43S COMPLEX | REACTOME | 52 | 0.003 |
| METABOLISM OF CARBOHYDRATES | REACTOME | 101 | 0.005 |
| CELL CYCLE CHECKPOINTS | REACTOME | 111 | 0.005 |
| GENE EXPRESSION | REACTOME | 351 | 0.008 |
| GLUCOSE METABOLISM | REACTOME | 77 | 0.008 |
| G1-S TRANSITION | REACTOME | 104 | 0.01 |
| ACTIVATION OF THE MRNA UPON BINDING OF THE CAP-BINDING COMPLEX AND EIFS, AND SUBSEQUENT BINDING TO 43S | REACTOME | 62 | 0.013 |
| ORC1 REMOVAL FROM CHROMATIN | REACTOME | 63 | 0.013 |
| DEGRADATION OF BETA-CATENIN BY THE DESTRUCTION COMPLEX | REACTOME | 56 | 0.014 |
| CYCLIN A:CDK2-ASSOCIATED EVENTS AT S PHASE ENTRY | REACTOME | 65 | 0.022 |
| DNA STRAND ELONGATION | REACTOME | 29 | 0.025 |
| AMINE-DERIVED HORMONES | REACTOME | 16 | 0.026 |
| NUCLEOTIDE METABOLISM | REACTOME | 82 | 0.028 |
| ELONGATION OF INTRON-CONTAINING TRANSCRIPTS AND CO-TRANSCRIPTIONAL MRNA SPLICING | REACTOME | 125 | 0.03 |
| ELONGATION AND PROCESSING OF CAPPED TRANSCRIPTS | REACTOME | 125 | 0.031 |
| APC-C:CDC20 MEDIATED DEGRADATION OF CYCLIN B | REACTOME | 31 | 0.032 |
| APC-CDC20 MEDIATED DEGRADATION OF NEK2A | REACTOME | 28 | 0.036 |
| CYCLIN E ASSOCIATED EVENTS DURING G1-S TRANSITION | REACTOME | 66 | 0.052 |
| FORMATION AND MATURATION OF MRNA TRANSCRIPT | REACTOME | 142 | 0.057 |
| ASSEMBLY OF THE RAD50-MRE11-NBS1 COMPLEX AT DNA DOUBLE-STRAND BREAKS | REACTOME | 19 | 0.057 |
| GLUCONEOGENESIS | REACTOME | 33 | 0.059 |
| G1-S DNA DAMAGE CHECKPOINTS | REACTOME | 54 | 0.062 |
| MITOTIC SPINDLE CHECKPOINT | REACTOME | 22 | 0.068 |
| ACTIVATION OF THE PRE-REPLICATIVE COMPLEX | REACTOME | 23 | 0.069 |
| MRN COMPLEX RELOCALIZES TO NUCLEAR FOCI | REACTOME | 19 | 0.07 |
| GAP-FILLING DNA REPAIR SYNTHESIS AND LIGATION IN TC-NER | REACTOME | 16 | 0.069 |
| FORMATION OF THE EARLY ELONGATION COMPLEX | REACTOME | 32 | 0.079 |
| CYTOSOLIC TRNA AMINOACYLATION | REACTOME | 27 | 0.082 |
| EXTENSION OF TELOMERES | REACTOME | 22 | 0.082 |
| INHIBITION OF THE PROTEOLYTIC ACTIVITY OF APC-C REQUIRED FOR THE ONSET OF ANAPHASE BY MITOTIC SPINDLE CHECKPOINT COMPONENTS | REACTOME | 21 | 0.082 |
| GLYCOLYSIS | REACTOME | 22 | 0.083 |
| ATM MEDIATED PHOSPHORYLATION OF REPAIR PROTEINS | REACTOME | 20 | 0.083 |
| GAP-FILLING DNA REPAIR SYNTHESIS AND LIGATION IN GG-NER | REACTOME | 16 | 0.084 |
| NEUROTRANSMITTER RELEASE CYCLE | REACTOME | 28 | 0.089 |
| INACTIVATION OF APC-C VIA DIRECT INHIBITION OF THE APC-C COMPLEX | REACTOME | 21 | 0.093 |
| CONVERSION FROM APC-C:CDC20 TO APC-C:CDH1 IN LATE ANAPHASE | REACTOME | 19 | 0.098 |
| LAGGING STRAND SYNTHESIS | REACTOME | 19 | 0.101 |
| ATM MEDIATED RESPONSE TO DNA DOUBLE-STRAND BREAK | REACTOME | 20 | 0.101 |
| PAUSING AND RECOVERY OF ELONGATION | REACTOME | 32 | 0.11 |
| ELONGATION ARREST AND RECOVERY | REACTOME | 32 | 0.111 |
| DNA REPAIR | REACTOME | 101 | 0.112 |
| FORMATION OF RNA POL II ELONGATION COMPLEX | REACTOME | 43 | 0.12 |
| NUCLEOTIDE EXCISION REPAIR | REACTOME | 50 | 0.122 |
| G2-M CHECKPOINTS | REACTOME | 39 | 0.129 |
| ATP FORMATION | REACTOME | 21 | 0.145 |
| HOMOLOGOUS RECOMBINATION REPAIR | REACTOME | 29 | 0.17 |
| HOMOLOGOUS RECOMBINATION REPAIR OF REPLICATION-INDEPENDENT DOUBLE-STRAND BREAKS | REACTOME | 29 | 0.169 |
| GENERATION OF SECOND MESSENGER MOLECULES | REACTOME | 22 | 0.173 |
| MITOCHONDRIAL TRNA AMINOACYLATION | REACTOME | 26 | 0.188 |
| ACTIVATION OF ATR IN RESPONSE TO REPLICATION STRESS | REACTOME | 29 | 0.195 |
| MITOTIC PROMETAPHASE | REACTOME | 90 | 0.212 |
| GLOBAL GENOMIC NER (GG-NER) | REACTOME | 34 | 0.216 |
| DOUBLE-STRAND BREAK REPAIR | REACTOME | 34 | 0.226 |
| M PHASE | REACTOME | 94 | 0.234 |
| METABOLISM OF NON-CODING RNA | REACTOME | 17 | 0.249 |

**Supplementary Figure legends**

**Supplementary Figure 1:** Kaplan-Meyer survival curves (A,C) and (B,D) Median survival from Kaplan-Meyer plots of *Rap1^+/+^Terc^+/+^*, *Rap1^-/-^Terc^+/+^,* G1 *Rap1^+/+^Terc^-/-^*, G1 *Rap1^-/-^Terc^-/-^*, G2 *Rap1^+/+^Terc^-/-^*, G2 *Rap1^-/-^Terc^-/-^,* G3 *Rap1^+/+^Terc^-/-^* and G3 *Rap1^-/-^Terc^-/-^* males (A,B) and females (C,D). The percent decrease in median survival in successive generations (G1, G2 and G3) of *Rap1^-/-^Terc^-/-^* compared to *Rap1^+/+^Terc^-/-^* mice is shown (red arrows). Statistical comparisons among genotypes using the log rank test (A,C) and Student t-test (B,D) are shown. (E) Body weight curves of *Rap1^+/+^Terc^+/+^*, *Rap1^-/-^Terc^+/+^,* G1 *Rap1^+/+^Terc^-/-^*, G1 *Rap1^-/-^Terc^-/-^*, G2 *Rap1^+/+^Terc^-/-^*, G2 *Rap1^-/-^Terc^-/-^,* G3 *Rap1^+/+^Terc^-/-^* and G3 *Rap1^-/-^Terc^-/-^* male mice. Values and error bars represent the mean and standard error, respectively. Statistical significances were calculated by the Studet t-test. (F) Body weight increment in RAP1 deficient females compared to WT controls determined at 10-week intervals throughout mouse lifespan. (n) Number of mice of each genotype used in the analysis

**Supplementary Figure 2:** (A) Incidence of kidney, lung, spleen and heart pathologies at death point of mice of the indicated genotypes. Kidney pathologies include glumerulonephritis, amyloidosis and lymphoid infiltrates. Lung pathology includes pneumonia. Spleen pathology includes hyperplasia. Heart pathologies include cardiac hypertrophy, myocardial infarction and calcifications (B) Incidence of liver pathologies in healthy young mice of the indicated genotype. Statistical significances were calculated by the Chi-squared test. Representative light microscopy images of hematoxylin-eosin stained sections of liver diagnosed for difused microvesicular and macrovesicular steatosis. (C) Mean telomere fluorescence intensity distribution in liver sections of healthy young mice of the indicated genotype as determined by Q-FISH analysis. (n) 4-5 mice per genotype were analyzed. The Student´s t-test was used for statistical analysis in each case.

**Supplementary Figure 3:** (A) Quantification of total cellular RAP1 levels in cell extract of two independent immortalized MEFS of the indicated genotypes by Western blot (WB). Representative WB images are shown. *Rap1* deleted MEFs were used as negative controls. SMC1 was used as loading control. (B) Autosomal genomic density of RAP1-binding sites expressed as occupancy per percentage of chromosome length, and ordered by ascending distance to the telomere in *Terc^+/+^* and in G1-G3 *Terc^-/-^* MEFs. A linear regression fit is shown for the three samples. RAP1 sites are enriched at the subtelomeric region in *Terc^+/+^* cells at all chromosomes (red dot) and the density decreases as the distance to the telomere increases. In G1-G3 *Terc^-/-^* the RAP1 peak density is more evenly distributed along the chromosome arms. A schemed representation a chromosome showing the telomere, the subtelomeric region and the centromere is depicted at the top.

**Supplementary Figure 4:** Disorders and diseases found transcriptionally deregulated (FDR<0.05) in (G0-G1-G3) RAP1-deficient MEFs as compared tp RAP1-proficient counterparts analyzed by Ingenuity software. Note similar pattern of affected disorders were observed in all the generations under study (G0-G1-G3).
